# The neglected puzzle of dementia in people with severe/profound intellectual disabilities: A systematic literature review of observable symptoms

**DOI:** 10.1111/jar.12920

**Published:** 2021-07-04

**Authors:** Maureen B. G. Wissing, Aurora M. Ulgiati, Johannes S. M. Hobbelen, Peter P. De Deyn, Aly Waninge, Alain D. Dekker

**Affiliations:** ^1^ Department of Neurology and Alzheimer Center University of Groningen, University Medical Center Groningen Groningen The Netherlands; ^2^ Research Group Healthy Ageing, Allied Health Care and Nursing Hanze University of Applied Sciences Groningen The Netherlands; ^3^ Department of Practice‐Oriented Scientific Research (PWO) Alliade Care Group Heerenveen The Netherlands; ^4^ Department of General Practice & Elderly Care Medicine University of Groningen, University Medical Center Groningen Groningen The Netherlands; ^5^ Institute Born‐Bunge University of Antwerpen Antwerp Belgium; ^6^ Department of Neurology and Memory Clinic Hospital Network Antwerp (ZNA) Middelheim and Hoge Beuken Antwerp Belgium; ^7^ Department of Health Psychology University of Groningen, University Medical Center Groningen Groningen The Netherlands; ^8^ Royal Dutch Visio Vries The Netherlands

**Keywords:** ageing, dementia, Down syndrome, intellectual disabilities, severe or profound intellectual (and multiple) disabilities

## Abstract

**Background:**

Dementia is increasingly prevalent in people with severe/profound intellectual disabilities. However, early detection and diagnosis of dementia is complex in this population. This study aimed to identify observable dementia symptoms in adults with severe/profound intellectual disabilities in available literature.

**Method:**

A systematic literature search was conducted in PubMed, PsycINFO and Web of Science with an exhaustive search string using a combination of search terms for severe/profound intellectual disabilities and dementia/ageing.

**Results:**

Eleven studies met inclusion criteria. Cognitive decline, behavioural and psychological alterations, decline in activities of daily living as well as neurological and physical changes were found.

**Conclusions:**

Only a very limited number of studies reported symptoms ascribed to dementia in adults with severe/profound intellectual disabilities. Given the complexity of signalling and diagnosing dementia, dedicated studies are required to unravel the natural history of dementia in this population.

## 1 INTRODUCTION

The world's population, including people with intellectual disabilities, is ageing. Life expectancy of people with intellectual disabilities has increased even more than in the general population (Coppus, 2013; Evans et al., 2013). Overall, life expectancy of this population is comparable to that of the general population, except for shorter life expectancy for people with more severe intellectual and multiple disabilities and people with Down syndrome (approximately 60 years; Bittles et al., 2007; Coppus, 2013). Given the fact that age is the major risk factor for dementia (Alzheimer's Association, 2021), dementia is increasingly prevalent among the population of people with disabilities. In particular, people with Down syndrome (trisomy 21) have a high genetic risk of developing Alzheimer's disease dementia: up to 77% will have developed dementia by the age between 60 and 69 years (Ballard et al., 2016). Moreover, prevalence rates of dementia for people with intellectual disabilities not due to Down syndrome vary across studies (Krinsky‐McHale & Silverman, 2013).

A pre‐existing intellectual disability, (lifelong) characteristic behaviour and co‐morbidities which may mimic dementia symptoms are complicating factors in diagnosing dementia in people with intellectual disabilities (Esbensen et al., 2017; McKenzie et al., 2018; Sheehan et al., 2015). In fact, the more severe the level of intellectual disability, the more difficult diagnosing dementia becomes (Evans et al., 2013). Hence, diagnosing dementia is especially challenging in people with severe/profound intellectual disabilities, that is, Intelligence Quotient score below 35 (Evans et al., 2013; McKenzie et al., 2018). Firstly, their low cognitive baseline functioning makes it difficult to establish a decline in cognitive functioning from a previous higher level (Evans et al., 2013). Secondly, to show measurable changes in cognitive functioning using direct neuropsychological tests is virtually impossible due to floor effects (Elliott‐King et al., 2016). Thirdly, observing a decline in functioning is highly complex because individuals with severe/profound intellectual disabilities have often multiple health conditions, that is, multimorbidity (Hermans & Evenhuis, 2014; Kinnear et al., 2018; Van Timmeren et al., 2017). Fourthly, they need high levels of support to perform activities of daily living, because specific skills were not attained (Sheehan et al., 2015). Consequently, skills which have never been developed cannot alter and therefore not serve as symptoms indicative of dementia. Lastly, diagnosing dementia in this population is even more complicated because communication is mostly non‐verbal, thus without self‐reported complaints (Smiley & Cooper, 2003). Hence, those with severe/profound intellectual disabilities are largely reliant on caregivers/relatives for signalling observable dementia symptoms (McKenzie et al., 2018).

Evidently, diagnosing dementia in people with severe/profound intellectual disabilities is a complex puzzle, which necessitates a proper understanding of its presentation in this population. Early detection and diagnosis of dementia allows care professionals and relatives to make informed choices about adaptation of caregiving, support and treatment (Dekker, Wissing et al., 2021). However, caregivers indicate to have limited knowledge on the presentation of dementia in people with intellectual disabilities (Herron et al., 2015; Whitehouse et al., 2000). Limited knowledge about symptoms may cause early signs not be recognised, resulting in a (too) late diagnosis or no diagnosis at all (Cleary & Doody, 2017). Moreover, if dementia was diagnosed there was a gap in intellectual disability caregivers' knowledge about the course of dementia (Furniss et al., 2011; Iacono et al., 2014). They struggled to understand whether changes were dementia symptoms or related to the intellectual disability (Iacono et al., 2014). Overall, a better understanding about (early) dementia symptoms, especially in those with severe/profound intellectual disabilities is essential to provide appropriate support and care in order to maintain quality of life (Janicki, 2011; Dekker, Wissing et al., 2021).

Improving the diagnostic procedure in this population starts with understanding the natural history of dementia. Hence, this study reviews literature to identify observable dementia symptoms in adults with severe/profound intellectual disabilities. Given the diagnostic complexity, it is expected that dementia is often underdiagnosed. Therefore, we also reviewed ageing literature describing changes in cognitive functioning and/or behavioural and psychological alterations without explicitly referring to dementia.

## 2 METHODS

This systematic literature review largely followed PRISMA criteria (Moher et al., 2009). All criteria were followed with the exception of a risk of bias assessment, since our core aim was to identify observable dementia symptoms in those with severe/profound intellectual disabilities in the scarce literature.

### 2.1 Search strategy

In December 2020, a systematic literature search without any time period restrictions was performed in PubMed, PsycINFO and Web of Science. The search strategy involved three key search term clusters. The first cluster included search terms for severe/profound intellectual disabilities, using a broad range of synonyms for intellectual disabilities as well as older (sometimes abandoned) terminology to ensure that relevant studies using past terminology were obtained as well. Given that no specific indexed terms exist for severe/profound intellectual disabilities in the databases, all search terms were searched in fivefold (preceded by the adjectives Complex, Multiple, Profound, Serious or Severe) to discard articles not focusing on severe/profound intellectual disabilities. The second cluster included search terms for dementia, for example, Alzheimer, major/minor cognitive impairment as well as terms related to ageing, such as decline, changes, progressive, deterioration. Subsequently, the third cluster ensured that only results for an aged population were obtained, removing large numbers of irrelevant studies in children, adolescents, young adults and animals. These three clusters were combined using the Boolean operator ‘AND’, whereas ‘OR’. In all three clusters, truncation (*) accounted for different forms of words. Terms were searched in title and abstract (Table A1).

### 2.2 Study selection

To be included, studies had to describe (potential) dementia symptoms in people with severe/profound intellectual disabilities aged 30 years and over. If studies focused on a broader spectrum of intellectual disability levels, (potential) dementia symptoms had to be separately reported for people with severe/profound intellectual disabilities. Exclusion criteria: studies in the general population (without intellectual disabilities), people with mild or moderate intellectual disabilities, age under 30 years, studies focusing on persons with severe/profound intellectual disabilities caused by rare (genetic) disorders (i.e., fewer than 5 in 10,000 people; Nguengang Wakap et al., 2020), non‐original research articles (e.g., reviews), animal studies and non‐English articles.

**FIGURE 1:**
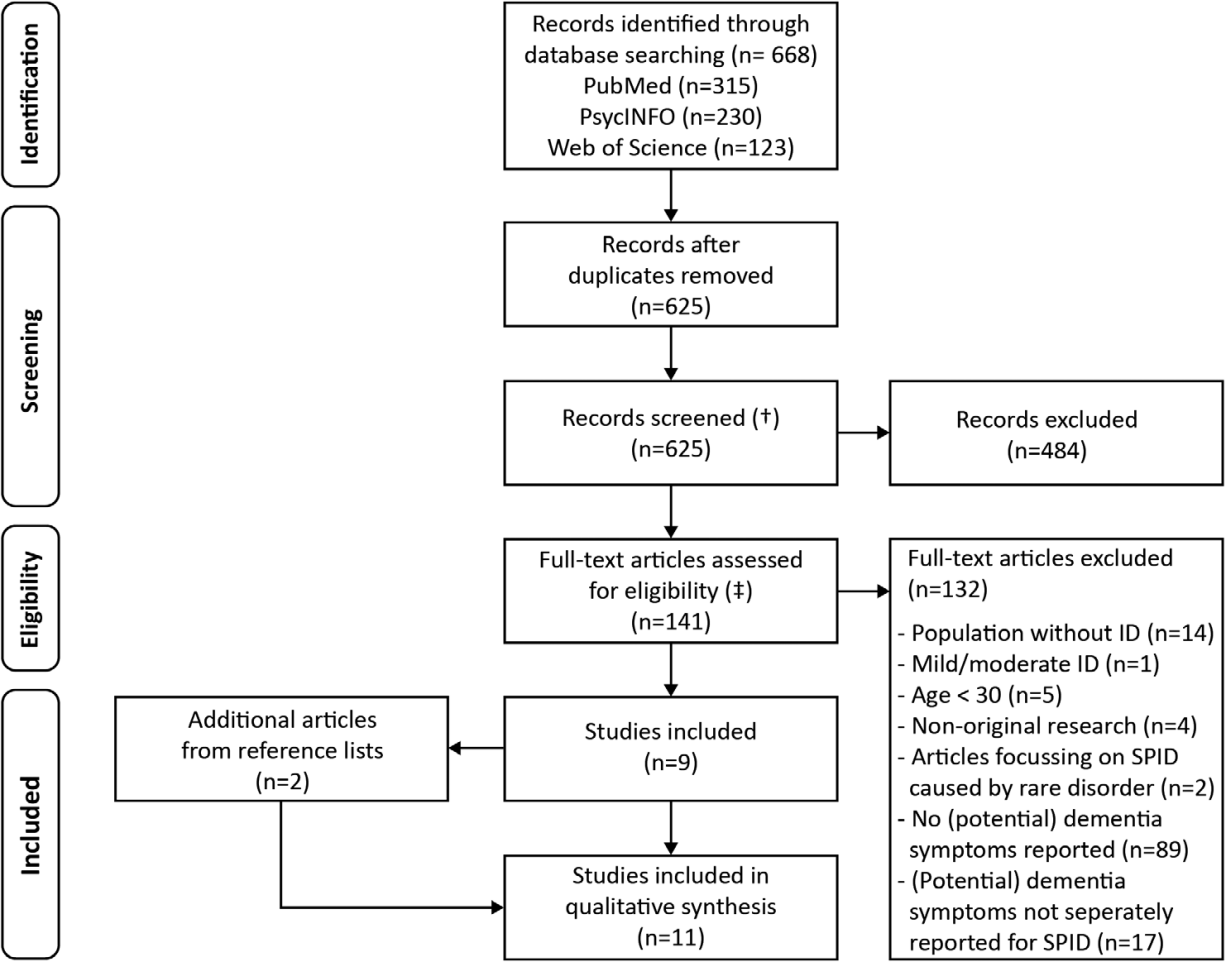
Flowchart of study selection process. †, inter‐rater agreement in 15% of deduplicated records; ‡, inter‐rater of full‐text screening. ID, intellectual disabilities; SPID, severe/profound intellectual disabilities

All records obtained in the three databases were deduplicated using RefWorks bibliographic management software (ProQuest). Titles and abstracts were subsequently screened for eligibility (A.M.U.). A randomly selected 15% of the deduplicated records were screened by a second author (M.B.G.W.). Afterwards, two authors (A.M.U. and M.B.G.W.) independently determined eligibility of the selected articles by checking full‐texts. Disagreements were resolved by consensus discussions, if necessary consulting a third author (A.D.D.). Lastly, reference lists of included studies were checked for additional articles. Figure 1 schematically depicts the selection procedure.

### 2.3 Data extraction and synthesis

Two authors (A.M.U. and M.B.G.W.) independently extracted relevant data from the selected full‐text studies, namely: study population(s), intellectual disability, assessment of dementia/age‐related changes that are potential dementia symptoms, main symptomatic results (Table 1). Discrepancies were resolved with discussions between the two authors. Additionally, two authors separately determined the limitations of the primary studies. To diagnose dementia, different sets of criteria are currently being used worldwide (American Psychiatric Association, 2013; McKhann et al., 2011; World Health Organization, 2018). Despite (minor) differences, these sets of criteria all include, in one way or another, a decline in cognitive functioning which interferes with the ability to perform activities of daily living, accompanied by behavioural and psychological symptoms of dementia (BPSD), (Dekker et al. 2015; Finkel, 2000). Therefore, in ageing studies only reported cognitive changes and behavioural and psychological alterations were considered to be potential dementia symptoms. In the present study, (potential) dementia symptoms were categorised according to the three diagnostic criteria domains. Additional findings reported in dementia studies were grouped in the category neurological and other physical changes (Nieuwenhuis‐Mark, 2009; Strydom et al., 2010; Table 2).

**TABLE 1:** Characteristics and main symptomatic results of included studies

	References	Study population(s)	ID classification	Assessment of dementia/age‐related changes that are potential dementia symptoms	Main symptomatic results
Studies of dementia symptoms	Reid and Aungle (1974)	155 ID, 63 ♂, 49–85 yrs ‐ 1 severe ID (DS) + dementia, ♂, 52 yrs ‐ 1 severe ID + dementia, ♀, 62 yrs	GMD	Clinical (re)assessment: ‐ Case files ‐ Informant interviews ‐ Physical examination	↓ activity, ↓ speech, ↓ self‐care skills, personality change, disturbed sleep, incontinence, late‐onset epilepsy (Case 1) ↓ self‐care skills, ↓ speech, personality change, asymmetrical spastic signs in limbs (Case 2)
	Day (1985)	357 ID, ≥ 40 yrs ‐ 2 severe ID + late‐onset dementia	ICD‐9	‐ Case files	↓ social skills, ↓ personal habits, behavioural disturbances, memory impairments: forgetfulness, confusion
	Evenhuis (1990)	17 DS, 7 ♂, 45–63 yrs ‐ 5 severe ID (DS) + AD, 2 ♂, 45–60 yrs	Severe ID: IQ 25–5	Non‐standardised clinical assessment: ‐ Observations ‐ Informant interviews ‐ Physical examination	↓ self‐help skills, ↓ gait, apathy/withdrawal, epileptic seizures, chair/bedridden (*n* = 5) urinary incontinence, daytime sleepiness, myoclonus (*n* = 4) disturbed night sleep, muscle hypertonia (*n* = 3) apraxia, irritability/aggression (*n* = 2) ↓ speech (*n* = 1)
	Duggan et al. (1996)	12 ID + dementia, 3 ♂, 47–77 yrs ‐ 1 severe ID (DS), ♀, 59 yrs	ICD‐9	‐ Psychiatric and medical files ‐ Informant interviews (BHI) ‐ Physical examination	↓ amount of walking, ↓ food intake, ↓ drinking, weight loss, inappropriate food placing, wrong utensils use, pica, aimless walking hypermetamorphosis,
	Burt et al. (1998)	70 DS, 22–60 yrs ‐ 2 severe ID (DS) + dementia ‐ 3 profound ID (DS) + dementia	Severe: IQ 21–36 Profound: no basal on LIPS	‐ Direct neuropsychological evaluation (DF & SR, GPT, DTVMI, PD, PPVT‐R, LIPS) ‐ Informant interviews (VABS, DQMRP, RSMB, DSI, MAS)	↓ cognitive functioning, ↓ memory, ↓ everyday functioning, emotional/behavioural changes
	Määttä et al. (2006)	129 DS, 76 ♂, 0–67 yrs ‐ 1 moderate/severe ID (DS) + AD, ♂, 51 yrs	ICD‐10	‐ Case files	↓ self‐care skills, ↑ forgetfulness, ↑ irritability, withdrawal, occasional aggressive outburst, late‐onset epilepsy
	Margallo‐Lana et al. (2007)	92 DS, 63 ♂, 20–76 yrs ‐ 6 profound ID (DS) + dementia	Unspecified	‐ Informant interviews ‐ Medical files	Non‐cognitive symptoms: ↓ everyday skills, ↓ mobility, ↓ interest in surroundings, uncharacteristic inappropriate behaviour, daytime sleepiness, wandering, getting lost, incontinence
	Sauna‐Aho et al. (2018)	128 DS, WS, FxS, 85 ♂, 36–85 yrs ‐ 2 profound ID + dementia, 50 yrs (DS), 61 yrs (FxS) ‐ 1 profound ID + vascular dementia, 53 yrs (WS)	Medical files	‐ Medical files ‐ BPSLD ‐ Brain imaging	Weight change, loss of energy, sleep disorder
Studies of potential dementia symptoms	Haveman et al. (1994)	1580 ID, 0–60+ yrs ‐ 209 severe ID (DS) ‐ 477 severe ID (non‐DS)	Unspecified	GQ: ‐ Psychological functioning ‐ Challenging behaviour	Non‐DS severe ID: = psychological problems, ↓ challenging behaviour DS severe ID: ↑ psychological problems, = challenging behaviour
	Cherry et al. (1997)	168 SPID ‐ 84 young, 46 ♂, 20–29 yrs ‐ 84 elderly, 45 ♂, 60–79 yrs	AAMD	DASH	Compared to younger SPID, older SPID showed: ↑ durations anxiety, inappropriate sexual behaviour, impulse control problems ↑ severity anxiety, stereotypies/tics, impulse control problems Result in relation to ID level: ↑ duration self‐injury behaviour in elderly with profound ID
	Rousseau et al. (2019)	474 profound ID and severe motor deficiency ‐ 219 young, 1.2 ♂/♀, 18–34 yrs ‐ 151 middle‐aged, 1 ♂/♀, 35–49 yrs ‐ 104 elderly, 1.4 ♂/♀, 50–68 yrs	Medical files	Medical files	↑ withdrawal, ↑ intermittent screaming, ↑ intermittent crying, ↑ agitation, ↑ self‐aggressivity, ↓ language, ↓ posture‐motor ability, ↓ coordination, ↓ sociability, = aggressivity, = stereotypies, = mericysm, = sleep disorders

*Note*: ↓, decrease; ↑, increase; =, remained stable, ♂, male; ♀, female.

Abbreviations: AAMD, American Association on Mental Deficiency; AD, Alzheimer's disease; DASH, Diagnostic Assessment for the Severely Handicapped; BHI, Behaviour History Inventory; BPSLD, British Present Psychiatric State‐Learning Disabilities assessment; DF & SR, Digits Forward and Sentence Recall subscales; DS, Down syndrome; DSI, Depression Status Inventory; DTVMI, Developmental Test of Visual‐Motor Integration; DQMRP, Dementia Questionnaire for Mentally Retarded Persons; FxS, Fragile X syndrome; GMD, Glossary of Mental Disorder; GPT, Grooved Pegboard Test; GQ, Gerontological Questionnaire subscales; ICD, International Classification of Diseases and Related Health Problems; ID, intellectual disabilities; IQ, Intelligence Quotient; LIPS, Leiter International Performance Scale; MAS, Mood Assessment Scale for MR; PD, Picture Discription test; PPVT‐R, Peabody Picture Vocabulary Test‐Revised; RSMB, Reiss Screen for Maladaptive Behaviour; SPID, severe/profound intellectual disabilities; VABS, Vineland Adaptive Behaviour Scale, WS, Williams syndrome; yrs, years.

**TABLE 2:** Overview of (potential) dementia symptoms in people with severe/profound intellectual disabilities

Categories	Dementia symptoms	Potential dementia symptoms
Cognitive changes	↓ speech^1,3^, ↓ social skills^2^, ↓ cognitive functioning^5^, ↓ memory^2,5^, forgetfulness^2,6^, confusion^2^, aimless walking^4^, wandering^7^, getting lost^7^, ↓ personal habits^2^, apraxia^3^, inappropriate food placing^4^, wrong utensils use^4^	↓ language^11^, ↓ sociability^11^, ↓ posture‐motor ability^11^, ↓ coordination^11^
Behavioural and psychological changes	aggression^3,6^, withdrawal^3,6^, apathy^3^,↓ interest in surroundings^7^, irritability^3,6^,daytime sleepiness^3,7^, disturbed sleep^1^, sleep disorder^8^, ↓ food intake^4^, ↓ drinking^4^, pica^4^, uncharacteristic inappropriate behaviour^7^, hypermetamorphosis^4^, personality change^1^, emotional/behavioural changes^5^	↑ self‐aggressivity^11^, ↑ duration self‐injury behaviour (PID)^10^, ↑ withdrawal^11^, ↑ agitation^11^, ↑ intermittent screaming^11^, ↑ intermittent crying^11^, ↑ durations anxiety^10^, ↑ durations inappropriate sexual behaviour^10^, ↑ durations impulse control problems^10^, ↑ severity anxiety^10^, ↑ severity stereotypies/tics^10^, ↑ severity impulse control problems^10^, ↑ psychological problems (DS)^9^
Changes in activities of daily living	↓ self‐care skills^1,3,6^, ↓ everyday functioning/skills^5,7^, ↓ activity^1^	
Neurological and other physical changes	incontinence^1,3,7^, (late‐onset) epilepsy^1,3,6^, weight change/loss^4,8^, ↓ energy^8^, ↓ amount of walking^4^, ↓ mobility^7^, ↓ gait^3^, chair/bedridden^3^, asymmetrical spastic signs in limbs^1^, muscle hypertonia^3^, myoclonus^3^	

*Note*: ↓, decrease; ↑, increase; =, remained stable. References: 1, (Reid & Aungle, 1974); 2, (Day, 1985); 3, (Evenhuis, 1990); 4, (Duggan et al., 1996); 5, (Burt et al., 1998); 6, (Määttä et al., 2006); 7, (Margallo‐Lana et al., 2007); 8, (Sauna‐Aho et al., 2018); 9, (Haveman et al., 1994); 10, (Cherry et al., 1997); 11, (Rousseau et al., 2019).

Abbreviations: DS, Down syndrome; non‐DS, without Down syndrome; PID, profound intellectual disabilities.

## 3 RESULTS

The literature search yielded a total of 668 hits in three databases. Deduplication resulted in 625 unique records (Figure 1). Based on title and abstract 141 records were considered potentially relevant for this review. Inter‐rater agreement of screening a randomly selected 15% of the deduplicated records was 96.7%. The 141 articles were read full‐text, 9 studies satisfied all criteria and were subsequently included. Inter‐rater agreement of this full‐text screening process was 96.2%. Two additional articles were identified by screening reference lists of included articles (Figure 1). In total, 11 studies met inclusion criteria. Table 1 provides a detailed overview of characteristics and main results.

### 3.1 Symptoms in dementia studies

The first study to report dementia symptoms in adults with severe/profound intellectual disabilities was published by Reid and Aungle (1974). Among 155 persons with intellectual disabilities, two individuals with a severe intellectual disability were diagnosed with dementia based on clinical (re)assessment (Table 1). For a 52‐year‐old man with Down syndrome, reported dementia symptoms consisted of a personality change, disturbed sleep, diminution of activity, reduction of speech and a deterioration in self‐care skills. Incontinence and late‐onset epilepsy were also reported. The second case concerned an ongoing dementia process in a 65‐year‐old woman with a severe intellectual disability of unknown aetiology. Dementia began in her middle 50s and signs were slowly progressive loss of self‐care skills, reduction of speech, a personality change and asymmetrical spasticity in the limbs.

Subsequently, Day (1985) reported the prevalence of dementia in 357 people with intellectual disabilities. A diagnosis of late‐onset dementia was recorded in case files of nine individuals, including two persons with a severe intellectual disability. Both individuals had memory impairments presented as forgetfulness and confusion as well as loss of social skills and deterioration in personal habits. Furthermore, they exhibited behavioural disturbances, which were not further specified for these two persons.

Evenhuis (1990) conducted a prospective longitudinal study in seventeen individuals with Down syndrome of whom five had a severe intellectual disability. Dementia was suspected in these five individuals based on progressive decline in activities of daily living. However, it was not possible to formally diagnose dementia because memory and orientation could not be evaluated. Decreased self‐help skills were observed in these five individuals as well. In one person, a reduction of speech was seen. In the other four persons speech had hardly developed, and was consequently not indicative of dementia (Table 1). Two persons had developed apraxia, whereas apraxia was not assessed in the other three persons. Additionally, apathy, for example, social withdrawal, was observed in all five participants. Furthermore, reported behavioural changes were daytime sleepiness (*n* = 4), disturbed sleep (*n* = 3) and irritability/aggression (*n* = 2). Resembling Reid and Aungle (1974) late‐onset of epileptic seizures (*n* = 5), myoclonus (*n* = 4) and incontinence (*n* = 4) were also reported. Additionally, all five individuals developed muscle hypertonia and presented gait deterioration. Over the course of dementia they became chair ridden or bedridden. Postmortem neuropathological examination confirmed Alzheimer's disease dementia in these five persons.

Duggan et al. (1996) described behavioural changes in a population with intellectual disabilities and dementia. Among the twelve individuals, one had a severe intellectual disability and was diagnosed with dementia. Based on an informant interview using the Past Behavioural History Inventory, this 59‐year‐old woman with Down syndrome was described as showing changes in walking behaviour, specifically aimless walking in the last eight months and a noticeable decrease in the amount of walking over the last eighteen months. Additional symptoms were weight loss, decreased food intake since eight months, inappropriate placing of food for eighteen months, wrong use of utensils, pica in the last six months and a decrease in the drinking amount over the last eight months. Lastly, she exhibited hypermetamorphosis which manifested as a compulsion to touch furniture (Table 1).

Similar to Evenhuis (1990), also Burt et al. (1998) used a prospective longitudinal approach for studying dementia in 70 people with Down syndrome, of whom at entry 16 had a severe intellectual disability and 5 a profound intellectual disability. Based on the International Classification of Diseases and Related Health Problems 10th revision, two persons with a severe intellectual disability and three with a profound intellectual disability had dementia (Table 1). A decline in memory was found in three persons and a decline in other cognitive functions was found in four individuals. In one person, direct memory and/or other cognitive tests were not possible, but decline was assumed to be present based on informant reports. All five persons declined in everyday functioning along with emotional and behavioural changes.

Furthermore, Määttä et al. (2006) focused on mental health and adaptive behaviour of 129 people with Down syndrome (Table 1). One case of an adult with a moderate–severe intellectual disability and Alzheimer's disease was described. Observations during the past five years revealed increasing forgetfulness, irritability, withdrawal, occasional aggressive outbursts and declining self‐care skills. In line with Reid and Aungle (1974), late‐onset epilepsy was found, here present for two and a half years.

Margallo‐Lana et al. (2007) studied the extent of cognitive changes and dementia in people with Down syndrome, emphasising that clinically diagnosing dementia in those with more severe intellectual disabilities could be problematic. For six persons with profound intellectual disabilities, the diagnosis of dementia was based on non‐cognitive dementia characteristics such as behavioural symptoms like loss of interest in surroundings, daytime sleepiness and uncharacteristic inappropriate behaviour. Further non‐cognitive signs were a decline in everyday skills, wandering and getting lost, as well as decreasing mobility and incontinence (Table 1).

The last study reporting dementia symptoms in people with severe/profound intellectual disabilities was published by Sauna‐Aho et al. (2018). Using the British Present and Psychiatric State‐learning Disabilities assessment dementia was screened in 128 individuals consisting of subjects with Down syndrome (*n* = 62), Williams syndrome (*n* = 22) and Fragile X syndrome (*n* = 44). A total of 50 individuals had a severe intellectual disability and 3 had a profound intellectual disability. However, specific dementia symptoms were only separately reported for the three individuals with profound intellectual disabilities (one Down syndrome, one Williams, one Fragile X syndrome), namely: weight change, loss of energy and sleep disorder (Table 1).

### 3.2 Potential dementia symptoms in ageing studies

It is expected that dementia is often underdiagnosed in people with severe/profound intellectual disabilities due to the complexity of diagnosing dementia in this population. Therefore, changes in cognitive functioning and/or behavioural and psychological alterations were considered to be potential symptoms of dementia.

The first study reporting age‐related changes, which can in fact be dementia symptoms in adults with severe/profound intellectual disabilities was published by Haveman et al. (1994). They evaluated challenging behaviour and psychological problems in 1580 persons with intellectual disabilities according to age, level of intellectual disability and presence of Down syndrome. Specifically for people with a severe intellectual disability without Down syndrome, they found less challenging behaviour with advanced age, whereas psychological problems were evenly distributed. In people with Down syndrome, elderly with severe intellectual disabilities had more psychological problems (Table 1). The authors concluded that these psychological problems can be explained as symptoms of dementia, given that 39 of the 85 persons with Down syndrome (mild and severe intellectual disabilities) aged 50 years or older had a diagnosis of dementia.

Next, Cherry et al. (1997) undertook a cross‐sectional study focusing on symptoms associated with psychiatric disorders in younger (20–29 years) versus older adults (60–79 years) with severe/profound intellectual disabilities (Table 1). Using the Diagnostic Assessment for the Severely Handicapped, psychopathologic symptoms were assessed based on frequency, duration and severity. Older adults showed longer durations for anxiety, inappropriate sexual behaviour and impulse control problems, as well as increased severity for anxiety, stereotypies/tics and impulse control problems. Moreover, the results implicated that anxiety and impulse control problems were more problematic in older adults. Additionally, age‐related changes in severe versus profound intellectual disabilities were compared. Older persons with a profound intellectual disability had longer durations of self‐injury behaviour compared to those with a severe intellectual disability. The authors reported that the prevalence of diagnosis for psychiatric disorders, particularly classic forms of mental illness like anxiety was low.

Lastly, Rousseau et al. (2019) evaluated ageing in 474 people with a profound intellectual disability and severe motor deficiency. Compared to younger individuals (18–34 years), older adults (50–68 years) presented more frequently behavioural problems like withdrawal, intermittent screaming, intermittent crying, agitation and self‐aggressivity. Similar proportions of aggressivity, stereotypies, mericysm and sleep problems were found in young, middle‐aged (35–49 years) and older persons. Moreover, cognitive skills including language, posture‐motor ability, coordination and sociability decreased with age.

### 3.3 Synthesis of results

In summary, eight studies reported dementia symptoms for in total 27 adults with severe/profound intellectual disabilities and dementia. Of these 27 individuals, 22 had Down syndrome 1 had Williams syndrome, 1 had Fragile X syndrome and for 3 individuals the aetiology was unspecified. Additionally, three studies focusing on age‐related changes in people with severe/profound intellectual disabilities found a decline in cognitive functioning and an increase of emergence of BPSD, which could potentially relate to dementia‐related symptoms given the complexity of diagnosing dementia in this population. Table 2 provides an overview of reported cognitive changes, BPSD, changes in the ability to perform activities of daily living, neurological and other physical changes.

### 3.4 Limitations of primary literature

The very limited number of studies that explicated studied dementia in people with severe/profound intellectual disabilities evidently showed that this population has been largely neglected in literature so far. Here, a first inventory of observable symptoms in this population is provided. Given that the retrieved articles had similar limitations, these limitations were not discussed per primary article but were summarised in general, grouped according to the data extraction categories presented in Table 1.

#### 3.4.1 Study population(s)

The first limitation concerned the small number of people with severe/profound intellectual disabilities for who observable dementia symptoms were reported (ranging from *n* = 1 to 6 in each dementia study). Secondly, the aetiology of the intellectual disability was not specified in two dementia studies for a total of three persons (Day, 1985; Reid & Aungle, 1974) as well as for the 477 persons without Down syndrome in the ageing study of Haveman et al. (1994) and 474 persons in the ageing study of Rousseau et al. (2019). Thirdly, the exact (sub)type of dementia was not reported in five dementia studies (Burt et al., 1998; Day, 1985; Duggan et al., 1996; Margallo‐Lana et al., 2007; Reid & Aungle, 1974). In another study it was not clearly reported whether the person with Down syndrome and the person with Fragile X syndrome had Alzheimer's disease dementia or vascular dementia Sauna‐Aho et al. (2018).

#### 3.4.2 Intellectual disability classification

Criteria to determine the level of intellectual disability varied across studies, introducing a potential degree of variation. In fact, two studies did not specify how the level of intellectual disability was established (Haveman et al., 1994; Margallo‐Lana et al., 2007). Surprisingly, Burt et al. (1998) determined the level at the entry of the study, introducing uncertainty about the premorbid level of intellectual disability, i.e., if those persons had always functioned in the severe/profound range at baseline.

#### 3.4.3 Assessment of dementia/age‐related changes that are potential dementia symptoms

Similar to determining the level of intellectual disability, assessment procedures for dementia in those with severe/profound intellectual disabilities varied across studies. Two studies obtained diagnoses from case files without elaborating on the exact diagnostic procedure (Day, 1985; Määttä et al., 2006). Five studies reported that they had used instruments to identify (potential) dementia symptoms (Burt et al., 1998; Cherry et al., 1997; Duggan et al., 1996; Haveman et al., 1994; Sauna‐Aho et al., 2018). In the remaining studies (potential) symptoms were retrieved from case files and/or information obtained from staff.

Taken together, studies retrieved in this systematic review displayed rather similar limitations with respect to small sample sizes and variation in assessment criteria/procedures.

## 4 DISCUSSION

To the best of our knowledge, this review is the first to systematically identify observable dementia symptoms in people with severe/profound intellectual disabilities and a clinically and/or postmortem confirmed diagnosis of dementia in the – very scarce – available literature. Using an extensive search strategy, only eight studies were identified focusing – in part – on dementia in this population. Additionally, given the complexity of diagnosing dementia, three studies describing a decline in cognitive functioning and/or behavioural and psychological alteration in the context of ageing were also included. Summarising symptoms of (potential) dementia, this review revealed a decline in cognitive functioning, involving a deterioration in speech and losses of social skills as well as BPSD, in particular withdrawal and aggressiveness. Furthermore, specifically in those with dementia, skills necessary to perform activities of daily living declined. Lastly, neurological and physical changes like, incontinence, (late‐onset) epilepsy and a deterioration in gait were reported.

In line with the diagnostic criteria of dementia, eight studies reported cognitive changes, merely for those with severe intellectual disabilities (American Psychiatric Association, 2013; McKhann et al., 2011; World Health Organization, 2018). Two studies reported that specifically in those with profound intellectual disabilities, it is virtually impossible to show measurable changes in cognitive function with neuropsychological tests (Evenhuis, 1990; Margallo‐Lana et al., 2007), which is in accordance with the findings of Elliott‐King et al. (2016). Cognitive functions may never have been acquired and therefore cannot decline (Holland et al., 2000). Furthermore, they have to be supported by care professionals for activities of daily living, making it complex to determine whether dementia‐related cognitive impairments interfere with the ability to perform activities of daily living. Nevertheless, it might be possible to determine dementia in this population based on BPSD, given that BPSD are found in all types of dementia and are most observable for caregivers (Engelborghs et al., 2005; Finkel, 2000).

In fact, the eight dementia studies as well as the three ageing studies all reported on BPSD. Four studies found symptoms of apathetic behaviour including withdrawal and loss of interest in surroundings (Evenhuis, 1990; Määttä et al., 2006; Margallo‐Lana et al., 2007; Rousseau et al., 2019). These results are consistent with recent findings in a large study on dementia in people with Down syndrome, in which apathy was found one of the most commonly observed BPSD symptoms (Dekker et al., 2021, 2018). Furthermore, in this study a substantial proportion of people with Down syndrome and Alzheimer's disease displayed an increased frequency of aggressive behaviour (Dekker et al., 2021, 2018). Similarly, two dementia studies included in this review reported aggression in three individuals with a severe intellectual disability, Down syndrome and dementia (Evenhuis, 1990; Määttä et al., 2006). Additionally, the prevalence of self‐aggressivity increased with age in people with a profound intellectual disability without an official diagnosis of dementia (Rousseau et al., 2019). Taken together, apathy and aggression reported in ageing studies can be signs of dementia in persons with severe/profound intellectual disabilities.

Furthermore, in those with a clinically and/or postmortem confirmed diagnosis of dementia, reported BPSD were irritability, alterations in eating/drinking behaviour and sleep problems. Irritability was observed particularly in individuals with a severe intellectual disability and Down syndrome (Evenhuis, 1990; Määttä et al., 2006), which is in line with results of other studies focusing on dementia symptoms in persons with Down syndrome (Lai & Williams, 1989; Moss & Patel, 1995). Moreover, Duggan et al. (1996) found alterations in eating and drinking behaviour also specifically in a person with a severe intellectual disability and Down syndrome. This suggests that eating and drinking behaviour is affected by dementia (Dekker et al., 2021, 2018). Additionally, individuals with severe/profound intellectual disabilities and dementia presented sleep problems including disturbed sleep and daytime sleepiness (Evenhuis, 1990; Reid & Aungle, 1974; Sauna‐Aho et al., 2018). Sleep problems are common in the population of people with intellectual disabilities (Van de Wouw et al., 2012). Nevertheless, they are important to consider given that they may aggravate cognitive decline and BPSD (Dekker et al., 2015).

Besides emerging BPSD, the ability to perform activities of daily living declined. Similar to Lai and Williams (1989), three dementia studies reported losses of self‐care skills in individuals with a severe intellectual disability and Down syndrome (Evenhuis, 1990; Määttä et al., 2006; Reid & Aungle, 1974). Furthermore, dementia studies reported symptoms related to neurological and other physical changes like, incontinence, (late‐onset) epilepsy, hypertonia and gait deterioration. In the study of Prasher (1995), these symptoms were associated with increasing severity of dementia.

### 4.1 Strengths

This systematic review is a first step towards a proper understanding of the presentation of dementia in the population of people with severe/profound intellectual disabilities. A thorough search strategy using a broad range of searching terms, including older (sometimes abandoned) terminology, was performed to identify studies reporting dementia symptoms. Besides, we reviewed ageing literature describing changes in cognitive functioning and/or behavioural and psychological alterations which can in fact be symptoms of dementia given the complexity of diagnosing dementia in this population. Indeed, in one ageing studies the authors confirmed that observed psychological problems were symptoms of dementia.

### 4.2 Limitations

Although this study provides the first steps towards a proper understanding of the natural history of dementia in people with severe/profound intellectual disabilities, various limitations were found across the primary literature retrieved here. The rather small number of people with severe/profound intellectual disabilities and dementia poses a threat to representativeness of these results for the entire population. Consequently, further analyses on aetiological subgroups were not possible (further complicated by evident lack of reporting of aetiologies). Establishing patterns of symptoms associated with different (sub)types of dementia could also not be determine because studies did not report the exact type of dementia.

Given the complexity of diagnosing dementia in people with severe/profound intellectual disabilities, it is questionable whether early dementia symptoms were observed or whether observed symptoms were attributed to the intellectual disability, ageing or another condition rather than dementia. For instance, cognitive changes and BPSD may also be caused by other causes than dementia, for example, sensory impairments and psychiatric disorders (Moriconi et al., 2015). In fact, studies reported high prevalence rates of visual or hearing deficits and psychiatric disorders including depression and schizophrenia in elderly with severe/profound intellectual disabilities (Evenhuis et al., 2001; Haveman & Maaskant, 1989; Kirkpatrick‐Sanchez et al., 1996; Van Splunder et al., 2006).

Furthermore, our search strategy was targeted to retrieve studies focusing on severe/profound intellectual disabilities. Given the functionalities of the databases PubMed, PsychINFO and Web of Science, studies assessing a broad level of intellectual disabilities without specifying the level in title or abstract were likely missed in the search strategy. To that end, we performed an additional search to assess how many studies focusing on dementia in people with intellectual disabilities (in the broadest sense) or persons with Down syndrome published in the last five years were potentially missed. No additional broad intellectual disability and Down syndrome studies reporting dementia symptoms in those with severe/profound intellectual disability were identified. This emphasises the lack of focus on dementia in this population.

### 4.3 Future implications

This systematic review provides a first overview of observable dementia symptoms in people with severe/profound intellectual disabilities. There is an evident need for further study of the natural history of dementia in this population. Future studies should focus on identification of observable dementia symptoms. Evidently, literature only provided limited clues. Therefore, it is of utmost importance to make an inventory of practice‐based observations, among others by analysing existing medical files and by collecting observed symptoms by care professionals with vast experience in intellectual disability care through surveys and interviews. Such information about symptomology of dementia is relevant to enable (early) detection and diagnosis of dementia in this population. This enables family and health care professionals to adequately adapt daily caregiving (Janicki, 2011; Dekker, Wissing et al., 2021). Furthermore, early diagnosis allows development of an individual treatment plan, including choices about medication use, to reduce specific symptoms or slow the rate of further decline (Janicki, 2011; Dekker, Wissing et al., 2021). Altogether, (early) diagnosis of dementia may contribute to the well‐being of individuals with severe/profound intellectual disabilities.

## 5 CONCLUSION

Dementia in people with severe/profound intellectual disabilities has received very little attention so far, as shown by the limited number of studies focusing on this complex combination. Here, we have identified and summarised observable symptoms in available literature. Despite the few small‐sized studies, a range of dementia symptoms were identified, subdivided in cognitive decline (e.g., memory loss, forgetfulness, deterioration in speech, losses of social skills), decline in activities of daily living (e.g., self‐cares skills, everyday functioning/skills), BPSD (e.g., apathy, aggression, irritability, altered eating/drinking behaviour) as well as neurologic and other physical symptoms (e.g., incontinence, (late‐onset) epilepsy, hypotonia, gait deterioration). Because of increasing life expectancy, dementia will become more prominent in people with severe/profound intellectual disabilities. This review is a first step in improving the diagnostic procedure in this population. Future studies are required to specifically address dementia in people with severe/profound intellectual disabilities, further establishing the natural history. This would enable (early) detection and diagnosis of dementia which contributes to maintaining quality of life in people with severe/profound intellectual disabilities and dementia.

## Appendix

**TABLE A1:** Search strategy for PubMed, PsycINFO and Web of Science

Database	Search strategy
PubMed	#1 Complex developmental abnormalit* [tiab] OR Multiple developmental abnormalit* [tiab] OR Profound developmental abnormalit* [tiab] OR Serious developmental abnormalit* [tiab] OR Severe developmental abnormalit* [tiab] OR Complex intellectual abnormalit* [tiab] OR Multiple intellectual abnormalit* [tiab] OR Profound intellectual abnormalit* [tiab] OR Serious intellectual abnormalit* [tiab] OR Severe intellectual abnormalit* [tiab] OR Complex learning abnormalit* [tiab] OR Multiple learning abnormalit* [tiab] OR Profound learning abnormalit* [tiab] OR Serious learning abnormalit* [tiab] OR Severe learning abnormalit* [tiab] OR Complex mental abnormalit* [tiab] OR Multiple mental abnormalit* [tiab] OR Profound mental abnormalit* [tiab] OR Serious mental abnormalit* [tiab] OR Severe mental abnormalit* [tiab] OR Complex neurodevelopmental abnormalit* [tiab] OR Multiple neurodevelopmental abnormalit* [tiab] OR Profound neurodevelopmental abnormalit* [tiab] OR Serious neurodevelopmental abnormalit* [tiab] OR Severe neurodevelopmental abnormalit* [tiab] OR amentia [tiab] OR Complex cognitive challenge* [tiab] OR Multiple cognitive challenge* [tiab] OR Profound cognitive challenge* [tiab] OR Serious cognitive challenge* [tiab] OR Severe cognitive challenge* [tiab] OR Complex developmental challenge* [tiab] OR Multiple developmental challenge* [tiab] OR Profound developmental challenge* [tiab] OR Serious developmental challenge* [tiab] OR Severe developmental challenge* [tiab] OR Complex intellectual challenge* [tiab] OR Multiple intellectual challenge* [tiab] OR Profound intellectual challenge* [tiab] OR Serious intellectual challenge* [tiab] OR Severe intellectual challenge* [tiab] OR Complex learning challenge* [tiab] OR Multiple learning challenge* [tiab] OR Profound learning challenge* [tiab] OR Serious learning challenge* [tiab] OR Severe learning challenge* [tiab] OR Complex neurodevelopmental challenge* [tiab] OR Multiple neurodevelopmental challenge* [tiab] OR Profound neurodevelopmental challenge* [tiab] OR Serious neurodevelopmental challenge* [tiab] OR Severe neurodevelopmental challenge* [tiab] OR Multiply cognitively challenged [tiab] OR Profoundly cognitively challenged [tiab] OR Seriously cognitively challenged [tiab] OR Severely cognitively challenged [tiab] OR Multiply developmentally challenged [tiab] OR Profoundly developmentally challenged [tiab] OR Seriously developmentally challenged [tiab] OR Severely developmentally challenged [tiab] OR Multiply intellectually challenged [tiab] OR Profoundly intellectually challenged [tiab] OR Seriously intellectually challenged [tiab] OR Severely intellectually challenged [tiab] OR Multiply learning challenged [tiab] OR Profoundly learning challenged [tiab] OR Seriously learning challenged [tiab] OR Severely learning challenged [tiab] OR Multiply neurodevelopmentally challenged [tiab] OR Profoundly neurodevelopmentally challenged [tiab] OR Seriously neurodevelopmentally challenged [tiab] OR Severely neurodevelopmentally challenged [tiab] OR Complex cognitive defect* [tiab] OR Multiple cognitive defect* [tiab] OR Profound cognitive defect* [tiab] OR Serious cognitive defect* [tiab] OR Severe cognitive defect* [tiab] OR Complex developmental defect* [tiab] OR Multiple developmental defect* [tiab] OR Profound developmental defect* [tiab] OR Serious developmental defect* [tiab] OR Severe developmental defect* [tiab] OR Complex intellectual defect* [tiab] OR Multiple intellectual defect* [tiab] OR Profound intellectual defect* [tiab] OR Serious intellectual defect* [tiab] OR Severe intellectual defect* [tiab] OR Complex learning defect* [tiab] OR Multiple learning defect* [tiab] OR Profound learning defect* [tiab] OR Serious learning defect* [tiab] OR Severe learning defect* [tiab] OR Complex mental defect* [tiab] OR Multiple mental defect* [tiab] OR Profound mental defect* [tiab] OR Serious mental defect* [tiab] OR Severe mental defect* [tiab] OR Complex neurodevelopmental defect* [tiab] OR Multiple neurodevelopmental defect* [tiab] OR Profound neurodevelopmental defect* [tiab] OR Serious neurodevelopmental defect* [tiab] OR Severe neurodevelopmental defect* [tiab] OR Multiply cognitively defective [tiab] OR Profoundly cognitively defective [tiab] OR Seriously cognitively defective [tiab] OR Severely cognitively defective [tiab] OR Multiply developmentally defective [tiab] OR Profoundly developmentally defective [tiab] OR Seriously developmentally defective [tiab] OR Severely developmentally defective [tiab] OR Multiply intellectually defective [tiab] OR Profoundly intellectually defective [tiab] OR Seriously intellectually defective [tiab] OR Severely intellectually defective [tiab] OR Multiply learning defective [tiab] OR Profoundly learning defective [tiab] OR Seriously learning defective [tiab] OR Severely learning defective [tiab] OR Multiply mentally defective [tiab] OR Profoundly mentally defective [tiab] OR Seriously mentally defective [tiab] OR Severely mentally defective [tiab] OR Multiply neurodevelopmentally defective [tiab] OR Profoundly neurodevelopmentally defective [tiab] OR Seriously neurodevelopmentally defective [tiab] OR Severely neurodevelopmentally defective [tiab] OR Complex cognitive deficienc* [tiab] OR Multiple cognitive deficienc* [tiab] OR Profound cognitive deficienc* [tiab] OR Serious cognitive deficienc* [tiab] OR Severe cognitive deficienc* [tiab] OR Complex developmental deficienc* [tiab] OR Multiple developmental deficienc* [tiab] OR Profound developmental deficienc* [tiab] OR Serious developmental deficienc* [tiab] OR Severe developmental deficienc* [tiab] OR Complex intellectual deficienc* [tiab] OR Multiple intellectual deficienc* [tiab] OR Profound intellectual deficienc* [tiab] OR Serious intellectual deficienc* [tiab] OR Severe intellectual deficienc* [tiab] OR Complex learning deficienc* [tiab] OR Multiple learning deficienc* [tiab] OR Profound learning deficienc* [tiab] OR Serious learning deficienc* [tiab] OR Severe learning deficienc* [tiab] OR Complex mental deficienc* [tiab] OR Multiple mental deficienc* [tiab] OR Profound mental deficienc* [tiab] OR Serious mental deficienc* [tiab] OR Severe mental deficienc* [tiab] OR Complex neurodevelopmental deficienc* [tiab] OR Multiple neurodevelopmental deficienc* [tiab] OR Profound neurodevelopmental deficienc* [tiab] OR Serious neurodevelopmental deficienc* [tiab] OR Severe neurodevelopmental deficienc* [tiab] OR Multiply cognitively deficient [tiab] OR Profoundly cognitively deficient [tiab] OR Seriously cognitively deficient [tiab] OR Severely cognitively deficient [tiab] OR Multiply developmentally deficient [tiab] OR Profoundly developmentally deficient [tiab] OR Seriously developmentally deficient [tiab] OR Severely developmentally deficient [tiab] OR Multiply intellectually deficient [tiab] OR Profoundly intellectually deficient [tiab] OR Seriously intellectually deficient [tiab] OR Severely intellectually deficient [tiab] OR Multiply learning deficient [tiab] OR Profoundly learning deficient [tiab] OR Seriously learning deficient [tiab] OR Severely learning deficient [tiab] OR Multiply mentally deficient [tiab] OR Profoundly mentally deficient [tiab] OR Seriously mentally deficient [tiab] OR Severely mentally deficient [tiab] OR Multiply neurodevelopmentally deficient [tiab] OR Profoundly neurodevelopmentally deficient [tiab] OR Seriously neurodevelopmentally deficient [tiab] OR Severely neurodevelopmentally deficient [tiab] OR Complex developmental deficit* [tiab] OR Multiple developmental deficit* [tiab] OR Profound developmental deficit* [tiab] OR Serious developmental deficit* [tiab] OR Severe developmental deficit* [tiab] OR Complex intellectual deficit* [tiab] OR Multiple intellectual deficit* [tiab] OR Profound intellectual deficit* [tiab] OR Serious intellectual deficit* [tiab] OR Severe intellectual deficit* [tiab] OR Complex learning deficit* [tiab] OR Multiple learning deficit* [tiab] OR Profound learning deficit* [tiab] OR Serious learning deficit* [tiab] OR Severe learning deficit* [tiab] OR Complex mental deficit* [tiab] OR Multiple mental deficit* [tiab] OR Profound mental deficit* [tiab] OR Serious mental deficit* [tiab] OR Severe mental deficit* [tiab] OR Complex neurodevelopmental deficit* [tiab] OR Multiple neurodevelopmental deficit* [tiab] OR Profound neurodevelopmental deficit* [tiab] OR Serious neurodevelopmental deficit* [tiab] OR Severe neurodevelopmental deficit* [tiab] OR Complex cognitive delay [tiab] OR Multiple cognitive delay [tiab] OR Profound cognitive delay [tiab] OR Serious cognitive delay [tiab] OR Severe cognitive delay [tiab] OR Complex developmental delay [tiab] OR Multiple developmental delay [tiab] OR Profound developmental delay [tiab] OR Serious developmental delay [tiab] OR Severe developmental delay [tiab] OR Complex intellectual delay [tiab] OR Multiple intellectual delay [tiab] OR Profound intellectual delay [tiab] OR Serious intellectual delay [tiab] OR Severe intellectual delay [tiab] OR Complex learning delay [tiab] OR Multiple learning delay [tiab] OR Profound learning delay [tiab] OR Serious learning delay [tiab] OR Severe learning delay [tiab] OR Complex mental delay [tiab] OR Multiple mental delay [tiab] OR Profound mental delay [tiab] OR Serious mental delay [tiab] OR Severe mental delay [tiab] OR Complex neurodevelopmental delay [tiab] OR Multiple neurodevelopmental delay [tiab] OR Profound neurodevelopmental delay [tiab] OR Serious neurodevelopmental delay [tiab] OR Severe neurodevelopmental delay [tiab] OR Multiply cognitively delayed [tiab] OR Profoundly cognitively delayed [tiab] OR Seriously cognitively delayed [tiab] OR Severely cognitively delayed [tiab] OR Multiply developmentally delayed [tiab] OR Profoundly developmentally delayed [tiab] OR Seriously developmentally delayed [tiab] OR Severely developmentally delayed [tiab] OR Multiply intellectually delayed [tiab] OR Profoundly intellectually delayed [tiab] OR Seriously intellectually delayed [tiab] OR Severely intellectually delayed [tiab] OR Multiply learning delayed [tiab] OR Profoundly learning delayed [tiab] OR Seriously learning delayed [tiab] OR Severely learning delayed [tiab] OR Multiply mentally delayed [tiab] OR Profoundly mentally delayed [tiab] OR Seriously mentally delayed [tiab] OR Severely mentally delayed [tiab] OR Multiply neurodevelopmentally delayed [tiab] OR Profoundly neurodevelopmentally delayed [tiab] OR Seriously neurodevelopmentally delayed [tiab] OR Severely neurodevelopmentally delayed [tiab] OR Multiply differently‐abled [tiab] OR Profoundly differently‐abled [tiab] OR Seriously differently‐abled [tiab] OR Severely differently‐abled [tiab] OR Multiple developmental difficult* [tiab] OR Complex developmental difficult* [tiab] OR Profound developmental difficult* [tiab] OR Serious developmental difficult* [tiab] OR Severe developmental difficult* [tiab] OR Multiple intellectual difficult* [tiab] OR Complex intellectual difficult* [tiab] OR Profound intellectual difficult* [tiab] OR Serious intellectual difficult* [tiab] OR Severe intellectual difficult* [tiab] OR Complex learning difficult* [tiab] OR Multiple learning difficult* [tiab] OR Profound learning difficult* [tiab] OR Serious learning difficult* [tiab] OR Severe learning difficult* [tiab] OR Multiple mental difficult* [tiab] OR Complex mental difficult* [tiab] OR Profound mental difficult* [tiab] OR Serious mental difficult* [tiab] OR Severe mental difficult* [tiab] OR Complex neurodevelopmental difficult* [tiab] OR Multiple neurodevelopmental difficult* [tiab] OR Profound neurodevelopmental difficult* [tiab] OR Serious neurodevelopmental difficult* [tiab] OR Severe neurodevelopmental difficult* [tiab] OR Complex cognitive disabilit* [tiab] OR Multiple cognitive disabilit* [tiab] OR Profound cognitive disabilit* [tiab] OR Serious cognitive disabilit* [tiab] OR Severe cognitive disabilit* [tiab] OR Complex developmental disabilit* [tiab] OR Multiple developmental disabilit* [tiab] OR Multiple developmental disabilit* [tiab] OR Serious developmental disabilit* [tiab] OR Severe developmental disabilit* [tiab] OR Trainable intellectual disabilit* [tiab] OR Complex intellectual disabilit* [tiab] OR Multiple intellectual disabilit* [tiab] OR Profound intellectual disabilit* [tiab] OR Serious intellectual disabilit* [tiab] OR Severe intellectual disabilit* [tiab] OR Severe profound intellectual motor disabilit* [tiab] OR Complex learning disabilit* [tiab] OR Multiple learning disabilit* [tiab] OR Profound learning disabilit* [tiab] OR Serious learning disabilit* [tiab] OR Severe learning disabilit* [tiab] OR Complex mental disabilit* [tiab] OR Multiple mental disabilit* [tiab] OR Profound mental disabilit* [tiab] OR Serious mental disabilit* [tiab] OR Severe mental disabilit* [tiab] OR Trainable mental disabilit* [tiab] OR Complex neurodevelopmental disabilit* [tiab] OR Multiple neurodevelopmental disabilit* [tiab] OR Profound neurodevelopmental disabilit* [tiab] OR Serious neurodevelopmental disabilit* [tiab] OR Severe neurodevelopmental disabilit* [tiab] OR Multiple disabilit* [tiab] OR Multiply cognitively disabled [tiab] OR Profoundly cognitively disabled [tiab] OR Seriously cognitively disabled [tiab] OR Severely cognitively disabled [tiab] OR Multiply developmentally disabled [tiab] OR Profoundly developmentally disabled [tiab] OR Seriously developmentally disabled [tiab] OR Severely developmentally disabled [tiab] OR Trainable developmentally disabled [tiab] OR Multiply intellectually disabled [tiab] OR Profoundly intellectually disabled [tiab] OR Seriously intellectually disabled [tiab] OR Severely intellectually disabled [tiab] OR Trainable intellectually disabled [tiab] OR Multiply learning disabled [tiab] OR Profoundly learning disabled [tiab] OR Seriously learning disabled [tiab] OR Severely learning disabled [tiab] OR Multiply mentally disabled [tiab] OR Profoundly mentally disabled [tiab] OR Seriously mentally disabled [tiab] OR Severely mentally disabled [tiab] OR Multiply neurodevelopmentally disabled [tiab] OR Profoundly neurodevelopmentally disabled [tiab] OR Seriously neurodevelopmentally disabled [tiab] OR Severely neurodevelopmentally disabled [tiab] OR Multiply disabled [tiab] OR Trainable mentally disabled [tiab] OR Complex developmental disorder* [tiab] OR Multiple developmental disorder* [tiab] OR Profound developmental disorder* [tiab] OR Serious developmental disorder* [tiab] OR Severe developmental disorder* [tiab] OR Complex intellectual disorder* [tiab] OR Multiple intellectual disorder* [tiab] OR Profound intellectual disorder* [tiab] OR Serious intellectual disorder* [tiab] OR Severe intellectual disorder* [tiab] OR Complex learning disorder* [tiab] OR Multiple learning disorder* [tiab] OR Profound learning disorder* [tiab] OR Serious learning disorder* [tiab] OR Severe learning disorder* [tiab] OR Complex neurodevelopmental disorder* [tiab] OR Multiple neurodevelopmental disorder* [tiab] OR Profound neurodevelopmental disorder* [tiab] OR Serious neurodevelopmental disorder* [tiab] OR Severe neurodevelopmental disorder* [tiab] OR Profoundly feeble‐minded [tiab] OR Seriously feeble‐minded [tiab] OR Severely feeble‐minded [tiab] OR Profound feeble‐mindedness [tiab] OR Serious feeble‐mindedness [tiab] OR Severe feeble‐mindedness [tiab] OR Complex cognitive handicap* [tiab] OR Multiple cognitive handicap* [tiab] OR Profound cognitive handicap* [tiab] OR Serious cognitive handicap* [tiab] OR Severe cognitive handicap* [tiab] OR Complex developmental handicap* [tiab] OR Multiple developmental handicap* [tiab] OR Profound developmental handicap* [tiab] OR Serious developmental handicap* [tiab] OR Severe developmental handicap* [tiab] OR Complex intellectual handicap* [tiab] OR Multiple intellectual handicap* [tiab] OR Profound intellectual handicap* [tiab] OR Serious intellectual handicap* [tiab] OR Severe intellectual handicap* [tiab] OR Complex learning handicap* [tiab] OR Multiple learning handicap* [tiab] OR Profound learning handicap* [tiab] OR Serious learning handicap* [tiab] OR Severe learning handicap* [tiab] OR Complex mental handicap* [tiab] OR Multiple mental handicap* [tiab] OR Profound mental handicap* [tiab] OR Serious mental handicap* [tiab] OR Severe mental handicap* [tiab] OR Trainable mental handicap* [tiab] OR Complex neurodevelopmental handicap* [tiab] OR Multiple neurodevelopmental handicap* [tiab] OR Profound neurodevelopmental handicap* [tiab] OR Serious neurodevelopmental handicap* [tiab] OR Severe neurodevelopmental handicap* [tiab] OR Multiply cognitively handicapped [tiab] OR Profoundly cognitively handicapped [tiab] OR Seriously cognitively handicapped [tiab] OR Severely cognitively handicapped [tiab] OR Multiply developmentally handicapped [tiab] OR Profoundly developmentally handicapped [tiab] OR Seriously developmentally handicapped [tiab] OR Severely developmentally handicapped [tiab] OR Multiply intellectually handicapped [tiab] OR Multiply intellectually handicapped [tiab] OR Profoundly intellectually handicapped [tiab] OR Profoundly intellectually handicapped [tiab] OR Seriously intellectually handicapped [tiab] OR Seriously intellectually handicapped [tiab] OR Severely intellectually handicapped [tiab] OR Severely intellectually handicapped [tiab] OR Multiply learning handicapped [tiab] OR Profoundly learning handicapped [tiab] OR Seriously learning handicapped [tiab] OR Severely learning handicapped [tiab] OR Multiply mentally handicapped [tiab] OR Profoundly mentally handicapped [tiab] OR Seriously mentally handicapped [tiab] OR Severely mentally handicapped [tiab] OR Multiply neurodevelopmentally handicapped [tiab] OR Profoundly neurodevelopmentally handicapped [tiab] OR Seriously neurodevelopmentally handicapped [tiab] OR Severely neurodevelopmentally handicapped [tiab] OR Multiply handicapped [tiab] OR Trainable mentally handicapped [tiab] OR idiocy [tiab] OR idiot [tiab] OR idiotcy [tiab] OR idiotic [tiab] OR idiotism [tiab] OR idiots [tiab] OR Imbecil* [tiab] OR Multiply developmentally impaired [tiab] OR Profoundly developmentally impaired [tiab] OR Seriously developmentally impaired [tiab] OR Severely developmentally impaired [tiab] OR Multiply intellectually impaired [tiab] OR Profoundly intellectually impaired [tiab] OR Seriously intellectually impaired [tiab] OR Severely intellectually impaired [tiab] OR Multiply learning impaired [tiab] OR Profoundly learning impaired [tiab] OR Seriously learning impaired [tiab] OR Severely learning impaired [tiab] OR Multiply mentally impaired [tiab] OR Profoundly mentally impaired [tiab] OR Seriously mentally impaired [tiab] OR Severely mentally impaired [tiab] OR Multiply neurodevelopmentally impaired [tiab] OR Profoundly neurodevelopmentally impaired [tiab] OR Seriously neurodevelopmentally impaired [tiab] OR Severely neurodevelopmentally impaired [tiab] OR Trainable mentally impaired [tiab] OR Complex developmental impairment* [tiab] OR Multiple developmental impairment* [tiab] OR Profound developmental impairment* [tiab] OR Serious developmental impairment* [tiab] OR Severe developmental impairment* [tiab] OR Complex intellectual impairment* [tiab] OR Multiple intellectual impairment* [tiab] OR Profound intellectual impairment* [tiab] OR Serious intellectual impairment* [tiab] OR Severe intellectual impairment* [tiab] OR Complex learning impairment* [tiab] OR Multiple learning impairment* [tiab] OR Profound learning impairment* [tiab] OR Serious learning impairment* [tiab] OR Severe learning impairment* [tiab] OR Complex mental impairment* [tiab] OR Multiple mental impairment* [tiab] OR Profound mental impairment* [tiab] OR Serious mental impairment* [tiab] OR Severe mental impairment* [tiab] OR Complex neurodevelopmental impairment* [tiab] OR Multiple neurodevelopmental impairment* [tiab] OR Profound neurodevelopmental impairment* [tiab] OR Serious neurodevelopmental impairment* [tiab] OR Severe neurodevelopmental impairment* [tiab] OR Trainable mental impairment* [tiab] OR Complex cognitive incapacit* [tiab] OR Multiple cognitive incapacit* [tiab] OR Profound cognitive incapacit* [tiab] OR Serious cognitive incapacit* [tiab] OR Severe cognitive incapacit* [tiab] OR Complex developmental incapacit* [tiab] OR Multiple developmental incapacit* [tiab] OR Profound developmental incapacit* [tiab] OR Serious developmental incapacit* [tiab] OR Severe developmental incapacit* [tiab] OR Complex intellectual incapacit* [tiab] OR Multiple intellectual incapacit* [tiab] OR Profound intellectual incapacit* [tiab] OR Serious intellectual incapacit* [tiab] OR Severe intellectual incapacit* [tiab] OR Complex learning incapacit* [tiab] OR Multiple learning incapacit* [tiab] OR Profound learning incapacit* [tiab] OR Serious learning incapacit* [tiab] OR Severe learning incapacit* [tiab] OR Complex mental incapacit* [tiab] OR Multiple mental incapacit* [tiab] OR Profound mental incapacit* [tiab] OR Serious mental incapacit* [tiab] OR Severe mental incapacit* [tiab] OR Complex neurodevelopmental incapacit* [tiab] OR Multiple neurodevelopmental incapacit* [tiab] OR Profound neurodevelopmental incapacit* [tiab] OR Serious neurodevelopmental incapacit* [tiab] OR Severe neurodevelopmental incapacit* [tiab] OR Multiply cognitively incapacitated [tiab] OR Profoundly cognitively incapacitated [tiab] OR Seriously cognitively incapacitated [tiab] OR Severely cognitively incapacitated [tiab] OR Multiply developmentally incapacitated [tiab] OR Profoundly developmentally incapacitated [tiab] OR Seriously developmentally incapacitated [tiab] OR Severely developmentally incapacitated [tiab] OR Multiply intellectually incapacitated [tiab] OR Profoundly intellectually incapacitated [tiab] OR Seriously intellectually incapacitated [tiab] OR Severely intellectually incapacitated [tiab] OR Multiply learning incapacitated [tiab] OR Profoundly learning incapacitated [tiab] OR Seriously learning incapacitated [tiab] OR Severely learning incapacitated [tiab] OR Multiply mentally incapacitated [tiab] OR Profoundly mentally incapacitated [tiab] OR Seriously mentally incapacitated [tiab] OR Severely mentally incapacitated [tiab] OR Multiply neurodevelopmentally incapacitated [tiab] OR Profoundly neurodevelopmentally incapacitated [tiab] OR Seriously neurodevelopmentally incapacitated [tiab] OR Severely neurodevelopmentally incapacitated [tiab] OR Polyhandicap* [tiab] OR Trainable mentally retardate* [tiab] OR Complex cognitive retardation [tiab] OR Multiple cognitive retardation [tiab] OR Profound cognitive retardation [tiab] OR Serious cognitive retardation [tiab] OR Severe cognitive retardation [tiab] OR Complex developmental retardation [tiab] OR Multiple developmental retardation [tiab] OR Profound developmental retardation [tiab] OR Serious developmental retardation [tiab] OR Severe developmental retardation [tiab] OR Complex intellectual retardation [tiab] OR Multiple intellectual retardation [tiab] OR Profound intellectual retardation [tiab] OR Serious intellectual retardation [tiab] OR Severe intellectual retardation [tiab] OR Complex learning retardation [tiab] OR Multiple learning retardation [tiab] OR Profound learning retardation [tiab] OR Serious learning retardation [tiab] OR Severe learning retardation [tiab] OR Complex mental retardation [tiab] OR Multiple mental retardation [tiab] OR Profound mental retardation [tiab] OR Serious mental retardation [tiab] OR Severe mental retardation [tiab] OR Trainable mental retardation [tiab] OR Complex neurodevelopmental retardation [tiab] OR Multiple neurodevelopmental retardation [tiab] OR Profound neurodevelopmental retardation [tiab] OR Serious neurodevelopmental retardation [tiab] OR Severe neurodevelopmental retardation [tiab] OR Multiply cognitively retarded [tiab] OR Profoundly cognitively retarded [tiab] OR Seriously cognitively retarded [tiab] OR Severely cognitively retarded [tiab] OR Multiply developmentally retarded [tiab] OR Profoundly developmentally retarded [tiab] OR Seriously developmentally retarded [tiab] OR Severely developmentally retarded [tiab] OR Multiply intellectually retarded [tiab] OR Profoundly intellectually retarded [tiab] OR Seriously intellectually retarded [tiab] OR Severely intellectually retarded [tiab] OR Multiply learning retarded [tiab] OR Profoundly learning retarded [tiab] OR Seriously learning retarded [tiab] OR Severely learning retarded [tiab] OR Multiply mentally retarded [tiab] OR Profoundly mentally retarded [tiab] OR Seriously mentally retarded [tiab] OR Severely mentally retarded [tiab] OR Multiply neurodevelopmentally retarded [tiab] OR Profoundly neurodevelopmentally retarded [tiab] OR Seriously neurodevelopmentally retarded [tiab] OR Severely neurodevelopmentally retarded [tiab] OR Trainable mentally retarded [tiab] OR Trainable retarded [tiab] OR Multiply cognitively subnormal [tiab] OR Profoundly cognitively subnormal [tiab] OR Seriously cognitively subnormal [tiab] OR Severely cognitively subnormal [tiab] OR Multiply developmentally subnormal [tiab] OR Profoundly developmentally subnormal [tiab] OR Seriously developmentally subnormal [tiab] OR Severely developmentally subnormal [tiab] OR Multiply intellectually subnormal [tiab] OR Profoundly intellectually subnormal [tiab] OR Seriously intellectually subnormal [tiab] OR Severely intellectually subnormal [tiab] OR Multiply learning subnormal [tiab] OR Profoundly learning subnormal [tiab] OR Seriously learning subnormal [tiab] OR Severely learning subnormal [tiab] OR Multiply mentally subnormal [tiab] OR Profoundly mentally subnormal [tiab] OR Seriously mentally subnormal [tiab] OR Severely mentally subnormal [tiab] OR Multiply neurodevelopmentally subnormal [tiab] OR Profoundly neurodevelopmentally subnormal [tiab] OR Seriously neurodevelopmentally subnormal [tiab] OR Severely neurodevelopmentally subnormal [tiab] OR Multiply weak‐minded [tiab] OR Profoundly weak‐minded [tiab] OR Seriously weak‐minded [tiab] OR Severely weak‐minded [tiab] OR Multiply weak‐mindedness [tiab] OR Profoundly weak‐mindedness [tiab] OR Seriously weak‐mindedness [tiab] OR Severely weak‐mindedness [tiab]) #2 Alzheimer* [tiab] OR Change [tiab] OR Changed [tiab] OR Changes [tiab] OR Changing [tiab] OR Decline [tiab] OR Dement* [tiab] OR Deteriorat* [tiab] OR Major cognitive dysfunction* [tiab] OR Major cognitive impairment* [tiab] OR Mild cognitive dysfunction* [tiab] OR Mild cognitive impairment* [tiab] OR Creutzfeldt‐Jakob [tiab] OR Degeneration [tiab] OR Neurodegenerat* [tiab] OR Kluver‐Bucy [tiab] OR Lewy Body [tiab] OR Cerebrovascular [tiab] OR Wilhelmsen Lynch [tiab] OR Pick* [tiab] OR Binswanger [tiab] OR Progressive [tiab] OR Leukoencephalopathy [tiab] OR Aging [tiab] OR Aging [tiab] OR Neurodeteriorat* [tiab] #3 Elder* [tiab] OR Geriatric* [tiab] OR Old age [tiab] OR Older participant* [tiab] OR Older population* [tiab] OR Older individual* [tiab] OR Older adult* [tiab] OR Older patient* [tiab] OR Older people [tiab] OR Older person* [tiab] OR Older subject* [tiab] OR Oldest participant* [tiab] OR Oldest population* [tiab] OR Oldest individual* [tiab] OR Oldest adult* [tiab] OR Oldest patient* [tiab] OR Oldest people [tiab] OR Oldest person* [tiab] OR Oldest subject* [tiab] OR Senior* [tiab] OR Aged individual* [tiab] OR Aged population* [tiab] OR Aged participant* [tiab] OR Aged adult* [tiab] OR Aged patient* [tiab] OR Aged people [tiab] OR Aged person* [tiab] OR Aged subject* [tiab] #4 #1 AND #2 AND #3
Web of Science	#1 “Complex developmental abnormalit*” OR “Multiple developmental abnormalit*” OR “Profound developmental abnormalit*” OR “Serious developmental abnormalit*” OR “Severe developmental abnormalit*” OR “Complex intellectual abnormalit*” OR “Multiple intellectual abnormalit*” OR “Profound intellectual abnormalit*” OR “Serious intellectual abnormalit*” OR “Severe intellectual abnormalit*” OR “Complex learning abnormalit*” OR “Multiple learning abnormalit*” OR “Profound learning abnormalit*” OR “Serious learning abnormalit*” OR “Severe learning abnormalit*” OR “Complex mental abnormalit*” OR “Multiple mental abnormalit*” OR “Profound mental abnormalit*” OR “Serious mental abnormalit*” OR “Severe mental abnormalit*” OR “Complex neurodevelopmental abnormalit*” OR “Multiple neurodevelopmental abnormalit*” OR “Profound neurodevelopmental abnormalit*” OR “Serious neurodevelopmental abnormalit*” OR “Severe neurodevelopmental abnormalit*” OR Amentia OR “Complex cognitive challenge*” OR “Multiple cognitive challenge*” OR “Profound cognitive challenge*” OR “Serious cognitive challenge*” OR “Severe cognitive challenge*” OR “Complex developmental challenge*” OR “Multiple developmental challenge*” OR “Profound developmental challenge*” OR “Serious developmental challenge*” OR “Severe developmental challenge*” OR “Complex intellectual challenge*” OR “Multiple intellectual challenge*” OR “Profound intellectual challenge*” OR “Serious intellectual challenge*” OR “Severe intellectual challenge*” OR “Complex learning challenge*” OR “Multiple learning challenge*” OR “Profound learning challenge*” OR “Serious learning challenge*” OR “Severe learning challenge*” OR “Complex neurodevelopmental challenge*” OR “Multiple neurodevelopmental challenge*” OR “Profound neurodevelopmental challenge*” OR “Serious neurodevelopmental challenge*” OR “Severe neurodevelopmental challenge*” OR “Multiply cognitively challenged” OR “Profoundly cognitively challenged” OR “Seriously cognitively challenged” OR “Severely cognitively challenged” OR “Multiply developmentally challenged” OR “Profoundly developmentally challenged” OR “Seriously developmentally challenged” OR “Severely developmentally challenged” OR “Multiply intellectually challenged” OR “Profoundly intellectually challenged” OR “Seriously intellectually challenged” OR “Severely intellectually challenged” OR “Multiply learning challenged” OR “Profoundly learning challenged” OR “Seriously learning challenged” OR “Severely learning challenged” OR “Multiply neurodevelopmentally challenged” OR “Profoundly neurodevelopmentally challenged” OR “Seriously neurodevelopmentally challenged” OR “Severely neurodevelopmentally challenged” OR “Complex cognitive defect*” OR “Multiple cognitive defect*” OR “Profound cognitive defect*” OR “Serious cognitive defect*” OR “Severe cognitive defect*” OR “Complex developmental defect*” OR “Multiple developmental defect*” OR “Profound developmental defect*” OR “Serious developmental defect*” OR “Severe developmental defect*” OR “Complex intellectual defect*” OR “Multiple intellectual defect*” OR “Profound intellectual defect*” OR “Serious intellectual defect*” OR “Severe intellectual defect*” OR “Complex learning defect*” OR “Multiple learning defect*” OR “Profound learning defect*” OR “Serious learning defect*” OR “Severe learning defect*” OR “Complex mental defect*” OR “Multiple mental defect*” OR “Profound mental defect*” OR “Serious mental defect*” OR “Severe mental defect*” OR “Complex neurodevelopmental defect*” OR “Multiple neurodevelopmental defect*” OR “Profound neurodevelopmental defect*” OR “Serious neurodevelopmental defect*” OR “Severe neurodevelopmental defect*” OR “Multiply cognitively defective” OR “Profoundly cognitively defective” OR “Seriously cognitively defective” OR “Severely cognitively defective” OR “Multiply developmentally defective” OR “Profoundly developmentally defective” OR “Seriously developmentally defective” OR “Severely developmentally defective” OR “Multiply intellectually defective” OR “Profoundly intellectually defective” OR “Seriously intellectually defective” OR “Severely intellectually defective” OR “Multiply learning defective” OR “Profoundly learning defective” OR “Seriously learning defective” OR “Severely learning defective” OR “Multiply mentally defective” OR “Profoundly mentally defective” OR “Seriously mentally defective” OR “Severely mentally defective” OR “Multiply neurodevelopmentally defective” OR “Profoundly neurodevelopmentally defective” OR “Seriously neurodevelopmentally defective” OR “Severely neurodevelopmentally defective” OR “Complex cognitive deficienc*” OR “Multiple cognitive deficienc*” OR “Profound cognitive deficienc*” OR “Serious cognitive deficienc*” OR “Severe cognitive deficienc*” OR “Complex developmental deficienc*” OR “Multiple developmental deficienc*” OR “Profound developmental deficienc*” OR “Serious developmental deficienc*” OR “Severe developmental deficienc*” OR “Complex intellectual deficienc*” OR “Multiple intellectual deficienc*” OR “Profound intellectual deficienc*” OR “Serious intellectual deficienc*” OR “Severe intellectual deficienc*” OR “Complex learning deficienc*” OR “Multiple learning deficienc*” OR “Profound learning deficienc*” OR “Serious learning deficienc*” OR “Severe learning deficienc*” OR “Complex mental deficienc*” OR “Multiple mental deficienc*” OR “Profound mental deficienc*” OR “Serious mental deficienc*” OR “Severe mental deficienc*” OR “Complex neurodevelopmental deficienc*” OR “Multiple neurodevelopmental deficienc*” OR “Profound neurodevelopmental deficienc*” OR “Serious neurodevelopmental deficienc*” OR “Severe neurodevelopmental deficienc*” OR “Multiply cognitively deficient” OR “Profoundly cognitively deficient” OR “Seriously cognitively deficient” OR “Severely cognitively deficient” OR “Multiply developmentally deficient” OR “Profoundly developmentally deficient” OR “Seriously developmentally deficient” OR “Severely developmentally deficient” OR “Multiply intellectually deficient” OR “Profoundly intellectually deficient” OR “Seriously intellectually deficient” OR “Severely intellectually deficient” OR “Multiply learning deficient” OR “Profoundly learning deficient” OR “Seriously learning deficient” OR “Severely learning deficient” OR “Multiply mentally deficient” OR “Profoundly mentally deficient” OR “Seriously mentally deficient” OR “Severely mentally deficient” OR “Multiply neurodevelopmentally deficient” OR “Profoundly neurodevelopmentally deficient” OR “Seriously neurodevelopmentally deficient” OR “Severely neurodevelopmentally deficient” OR “Complex developmental deficit*” OR “Multiple developmental deficit*” OR “Profound developmental deficit*” OR “Serious developmental deficit*” OR “Severe developmental deficit*” OR “Complex intellectual deficit*” OR “Multiple intellectual deficit*” OR “Profound intellectual deficit*” OR “Serious intellectual deficit*” OR “Severe intellectual deficit*” OR “Complex learning deficit*” OR “Multiple learning deficit*” OR “Profound learning deficit*” OR “Serious learning deficit*” OR “Severe learning deficit*” OR “Complex mental deficit*” OR “Multiple mental deficit*” OR “Profound mental deficit*” OR “Serious mental deficit*” OR “Severe mental deficit*” OR “Complex neurodevelopmental deficit*” OR “Multiple neurodevelopmental deficit*” OR “Profound neurodevelopmental deficit*” OR “Serious neurodevelopmental deficit*” OR “Severe neurodevelopmental deficit*” OR “Complex cognitive delay” OR “Multiple cognitive delay” OR “Profound cognitive delay” OR “Serious cognitive delay” OR “Severe cognitive delay” OR “Complex developmental delay” OR “Multiple developmental delay” OR “Profound developmental delay” OR “Serious developmental delay” OR “Severe developmental delay” OR “Complex intellectual delay” OR “Multiple intellectual delay” OR “Profound intellectual delay” OR “Serious intellectual delay” OR “Severe intellectual delay” OR “Complex learning delay” OR “Multiple learning delay” OR “Profound learning delay” OR “Serious learning delay” OR “Severe learning delay” OR “Complex mental delay” OR “Multiple mental delay” OR “Profound mental delay” OR “Serious mental delay” OR “Severe mental delay” OR “Complex neurodevelopmental delay” OR “Multiple neurodevelopmental delay” OR “Profound neurodevelopmental delay” OR “Serious neurodevelopmental delay” OR “Severe neurodevelopmental delay” OR “Multiply cognitively delayed” OR “Profoundly cognitively delayed” OR “Seriously cognitively delayed” OR “Severely cognitively delayed” OR “Multiply developmentally delayed” OR “Profoundly developmentally delayed” OR “Seriously developmentally delayed” OR “Severely developmentally delayed” OR “Multiply intellectually delayed” OR “Profoundly intellectually delayed” OR “Seriously intellectually delayed” OR “Severely intellectually delayed” OR “Multiply learning delayed” OR “Profoundly learning delayed” OR “Seriously learning delayed” OR “Severely learning delayed” OR “Multiply mentally delayed” OR “Profoundly mentally delayed” OR “Seriously mentally delayed” OR “Severely mentally delayed” OR “Multiply neurodevelopmentally delayed” OR “Profoundly neurodevelopmentally delayed” OR “Seriously neurodevelopmentally delayed” OR “Severely neurodevelopmentally delayed” OR “Multiply differently‐abled” OR “Profoundly differently‐abled” OR “Seriously differently‐abled” OR “Severely differently‐abled” OR “Multiple developmental difficult*” OR “Complex developmental difficult*” OR “Profound developmental difficult*” OR “Serious developmental difficult*” OR “Severe developmental difficult*” OR “Multiple intellectual difficult*” OR “Complex intellectual difficult*” OR “Profound intellectual difficult*” OR “Serious intellectual difficult*” OR “Severe intellectual difficult*” OR “Complex learning difficult*” OR “Multiple learning difficult*” OR “Profound learning difficult*” OR “Serious learning difficult*” OR “Severe learning difficult*” OR “Multiple mental difficult*” OR “Complex mental difficult*” OR “Profound mental difficult*” OR “Serious mental difficult*” OR “Severe mental difficult*” OR “Complex neurodevelopmental difficult*” OR “Multiple neurodevelopmental difficult*” OR “Profound neurodevelopmental difficult*” OR “Serious neurodevelopmental difficult*” OR “Severe neurodevelopmental difficult*” OR “Complex cognitive disabilit*” OR “Multiple cognitive disabilit*” OR “Profound cognitive disabilit*” OR “Serious cognitive disabilit*” OR “Severe cognitive disabilit*” OR “Complex developmental disabilit*” OR “Multiple developmental disabilit*” OR “Multiple developmental disabilit*” OR “Serious developmental disabilit*” OR “Severe developmental disabilit*” OR “Trainable intellectual disabilit*” OR “Complex intellectual disabilit*” OR “Multiple intellectual disabilit*” OR “Profound intellectual disabilit*” OR “Serious intellectual disabilit*” OR “Severe intellectual disabilit*” OR “Severe profound intellectual motor disabilit*” OR “Complex learning disabilit*” OR “Multiple learning disabilit*” OR “Profound learning disabilit*” OR “Serious learning disabilit*” OR “Severe learning disabilit*” OR “Complex mental disabilit*” OR “Multiple mental disabilit*” OR “Profound mental disabilit*” OR “Serious mental disabilit*” OR “Severe mental disabilit*” OR “Trainable mental disabilit*” OR “Complex neurodevelopmental disabilit*” OR “Multiple neurodevelopmental disabilit*” OR “Profound neurodevelopmental disabilit*” OR “Serious neurodevelopmental disabilit*” OR “Severe neurodevelopmental disabilit*” OR “Multiple disabilit*” OR “Multiply cognitively disabled” OR “Profoundly cognitively disabled” OR “Seriously cognitively disabled” OR “Severely cognitively disabled” OR “Multiply developmentally disabled” OR “Profoundly developmentally disabled” OR “Seriously developmentally disabled” OR “Severely developmentally disabled” OR “Trainable developmentally disabled” OR “Multiply intellectually disabled” OR “Profoundly intellectually disabled” OR “Seriously intellectually disabled” OR “Severely intellectually disabled” OR “Trainable intellectually disabled” OR “Multiply learning disabled” OR “Profoundly learning disabled” OR “Seriously learning disabled” OR “Severely learning disabled” OR “Multiply mentally disabled” OR “Profoundly mentally disabled” OR “Seriously mentally disabled” OR “Severely mentally disabled” OR “Multiply neurodevelopmentally disabled” OR “Profoundly neurodevelopmentally disabled” OR “Seriously neurodevelopmentally disabled” OR “Severely neurodevelopmentally disabled” OR “Multiply disabled” OR “Trainable mentally disabled” OR “Complex developmental disorder*” OR “Multiple developmental disorder*” OR “Profound developmental disorder*” OR “Serious developmental disorder*” OR “Severe developmental disorder*” OR “Complex intellectual disorder*” OR “Multiple intellectual disorder*” OR “Profound intellectual disorder*” OR “Serious intellectual disorder*” OR “Severe intellectual disorder*” OR “Complex learning disorder*” OR “Multiple learning disorder*” OR “Profound learning disorder*” OR “Serious learning disorder*” OR “Severe learning disorder*” OR “Complex neurodevelopmental disorder*” OR “Multiple neurodevelopmental disorder*” OR “Profound neurodevelopmental disorder*” OR “Serious neurodevelopmental disorder*” OR “Severe neurodevelopmental disorder*” OR “Profoundly feeble‐minded” OR “Seriously feeble‐minded” OR “Severely feeble‐minded” OR “Profound feeble‐mindedness” OR “Serious feeble‐mindedness” OR “Severe feeble‐mindedness” OR “Complex cognitive handicap*” OR “Multiple cognitive handicap*” OR “Profound cognitive handicap*” OR “Serious cognitive handicap*” OR “Severe cognitive handicap*” OR “Complex developmental handicap*” OR “Multiple developmental handicap*” OR “Profound developmental handicap*” OR “Serious developmental handicap*” OR “Severe developmental handicap*” OR “Complex intellectual handicap*” OR “Multiple intellectual handicap*” OR “Profound intellectual handicap*” OR “Serious intellectual handicap*” OR “Severe intellectual handicap*” OR “Complex learning handicap*” OR “Multiple learning handicap*” OR “Profound learning handicap*” OR “Serious learning handicap*” OR “Severe learning handicap*” OR “Complex mental handicap*” OR “Multiple mental handicap*” OR “Profound mental handicap*” OR “Serious mental handicap*” OR “Severe mental handicap*” OR “Trainable mental handicap*” OR “Complex neurodevelopmental handicap*” OR “Multiple neurodevelopmental handicap*” OR “Profound neurodevelopmental handicap*” OR “Serious neurodevelopmental handicap*” OR “Severe neurodevelopmental handicap*” OR “Multiply cognitively handicapped” OR “Profoundly cognitively handicapped” OR “Seriously cognitively handicapped” OR “Severely cognitively handicapped” OR “Multiply developmentally handicapped” OR “Profoundly developmentally handicapped” OR “Seriously developmentally handicapped” OR “Severely developmentally handicapped” OR “Multiply intellectually handicapped” OR “Multiply intellectually handicapped” OR “Profoundly intellectually handicapped” OR “Profoundly intellectually handicapped” OR “Seriously intellectually handicapped” OR “Seriously intellectually handicapped” OR “Severely intellectually handicapped” OR “Severely intellectually handicapped” OR “Multiply learning handicapped” OR “Profoundly learning handicapped” OR “Seriously learning handicapped” OR “Severely learning handicapped” OR “Multiply mentally handicapped” OR “Profoundly mentally handicapped” OR “Seriously mentally handicapped” OR “Severely mentally handicapped” OR “Multiply neurodevelopmentally handicapped” OR “Profoundly neurodevelopmentally handicapped” OR “Seriously neurodevelopmentally handicapped” OR “Severely neurodevelopmentally handicapped” OR “Multiply handicapped” OR “Trainable mentally handicapped” OR Idiocy OR Idiot OR Idiotcy OR Idiotic OR Idiotism OR Idiots OR Imbecil* OR “Multiply developmentally impaired” OR “Profoundly developmentally impaired” OR “Seriously developmentally impaired” OR “Severely developmentally impaired” OR “Multiply intellectually impaired” OR “Profoundly intellectually impaired” OR “Seriously intellectually impaired” OR “Severely intellectually impaired” OR “Multiply learning impaired” OR “Profoundly learning impaired” OR “Seriously learning impaired” OR “Severely learning impaired” OR “Multiply mentally impaired” OR “Profoundly mentally impaired” OR “Seriously mentally impaired” OR “Severely mentally impaired” OR “Multiply neurodevelopmentally impaired” OR “Profoundly neurodevelopmentally impaired” OR “Seriously neurodevelopmentally impaired” OR “Severely neurodevelopmentally impaired” OR “Trainable mentally impaired” OR “Complex developmental impairment*” OR “Multiple developmental impairment*” OR “Profound developmental impairment*” OR “Serious developmental impairment*” OR “Severe developmental impairment*” OR “Complex intellectual impairment*” OR “Multiple intellectual impairment*” OR “Profound intellectual impairment*” OR “Serious intellectual impairment*” OR “Severe intellectual impairment*” OR “Complex learning impairment*” OR “Multiple learning impairment*” OR “Profound learning impairment*” OR “Serious learning impairment*” OR “Severe learning impairment*” OR “Complex mental impairment*” OR “Multiple mental impairment*” OR “Profound mental impairment*” OR “Serious mental impairment*” OR “Severe mental impairment*” OR “Complex neurodevelopmental impairment*” OR “Multiple neurodevelopmental impairment*” OR “Profound neurodevelopmental impairment*” OR “Serious neurodevelopmental impairment*” OR “Severe neurodevelopmental impairment*” OR “Trainable mental impairment*” OR “Complex cognitive incapacit*” OR “Multiple cognitive incapacit*” OR “Profound cognitive incapacit*” OR “Serious cognitive incapacit*” OR “Severe cognitive incapacit*” OR “Complex developmental incapacit*” OR “Multiple developmental incapacit*” OR “Profound developmental incapacit*” OR “Serious developmental incapacit*” OR “Severe developmental incapacit*” OR “Complex intellectual incapacit*” OR “Multiple intellectual incapacit*” OR “Profound intellectual incapacit*” OR “Serious intellectual incapacit*” OR “Severe intellectual incapacit*” OR “Complex learning incapacit*” OR “Multiple learning incapacit*” OR “Profound learning incapacit*” OR “Serious learning incapacit*” OR “Severe learning incapacit*” OR “Complex mental incapacit*” OR “Multiple mental incapacit*” OR “Profound mental incapacit*” OR “Serious mental incapacit*” OR “Severe mental incapacit*” OR “Complex neurodevelopmental incapacit*” OR “Multiple neurodevelopmental incapacit*” OR “Profound neurodevelopmental incapacit*” OR “Serious neurodevelopmental incapacit*” OR “Severe neurodevelopmental incapacit*” OR “Multiply cognitively incapacitated” OR “Profoundly cognitively incapacitated” OR “Seriously cognitively incapacitated” OR “Severely cognitively incapacitated” OR “Multiply developmentally incapacitated” OR “Profoundly developmentally incapacitated” OR “Seriously developmentally incapacitated” OR “Severely developmentally incapacitated” OR “Multiply intellectually incapacitated” OR “Profoundly intellectually incapacitated” OR “Seriously intellectually incapacitated” OR “Severely intellectually incapacitated” OR “Multiply learning incapacitated” OR “Profoundly learning incapacitated” OR “Seriously learning incapacitated” OR “Severely learning incapacitated” OR “Multiply mentally incapacitated” OR “Profoundly mentally incapacitated” OR “Seriously mentally incapacitated” OR “Severely mentally incapacitated” OR “Multiply neurodevelopmentally incapacitated” OR “Profoundly neurodevelopmentally incapacitated” OR “Seriously neurodevelopmentally incapacitated” OR “Severely neurodevelopmentally incapacitated” OR “Polyhandicap*” OR “Trainable mentally retardate*” OR “Complex cognitive retardation” OR “Multiple cognitive retardation” OR “Profound cognitive retardation” OR “Serious cognitive retardation” OR “Severe cognitive retardation” OR “Complex developmental retardation” OR “Multiple developmental retardation” OR “Profound developmental retardation” OR “Serious developmental retardation” OR “Severe developmental retardation” OR “Complex intellectual retardation” OR “Multiple intellectual retardation” OR “Profound intellectual retardation” OR “Serious intellectual retardation” OR “Severe intellectual retardation” OR “Complex learning retardation” OR “Multiple learning retardation” OR “Profound learning retardation” OR “Serious learning retardation” OR “Severe learning retardation” OR “Complex mental retardation” OR “Multiple mental retardation” OR “Profound mental retardation” OR “Serious mental retardation” OR “Severe mental retardation” OR “Trainable mental retardation” OR “Complex neurodevelopmental retardation” OR “Multiple neurodevelopmental retardation” OR “Profound neurodevelopmental retardation” OR “Serious neurodevelopmental retardation” OR “Severe neurodevelopmental retardation” OR “Multiply cognitively retarded” OR “Profoundly cognitively retarded” OR “Seriously cognitively retarded” OR “Severely cognitively retarded” OR “Multiply developmentally retarded” OR “Profoundly developmentally retarded” OR “Seriously developmentally retarded” OR “Severely developmentally retarded” OR “Multiply intellectually retarded” OR “Profoundly intellectually retarded” OR “Seriously intellectually retarded” OR “Severely intellectually retarded” OR “Multiply learning retarded” OR “Profoundly learning retarded” OR “Seriously learning retarded” OR “Severely learning retarded” OR “Multiply mentally retarded” OR “Profoundly mentally retarded” OR “Seriously mentally retarded” OR “Severely mentally retarded” OR “Multiply neurodevelopmentally retarded” OR “Profoundly neurodevelopmentally retarded” OR “Seriously neurodevelopmentally retarded” OR “Severely neurodevelopmentally retarded” OR “Trainable mentally retarded” OR “Trainable retarded” OR “Multiply cognitively subnormal” OR “Profoundly cognitively subnormal” OR “Seriously cognitively subnormal” OR “Severely cognitively subnormal” OR “Multiply developmentally subnormal” OR “Profoundly developmentally subnormal” OR “Seriously developmentally subnormal” OR “Severely developmentally subnormal” OR “Multiply intellectually subnormal” OR “Profoundly intellectually subnormal” OR “Seriously intellectually subnormal” OR “Severely intellectually subnormal” OR “Multiply learning subnormal” OR “Profoundly learning subnormal” OR “Seriously learning subnormal” OR “Severely learning subnormal” OR “Multiply mentally subnormal” OR “Profoundly mentally subnormal” OR “Seriously mentally subnormal” OR “Severely mentally subnormal” OR “Multiply neurodevelopmentally subnormal” OR “Profoundly neurodevelopmentally subnormal” OR “Seriously neurodevelopmentally subnormal” OR “Severely neurodevelopmentally subnormal” OR “Multiply weak‐minded” OR “Profoundly weak‐minded” OR “Seriously weak‐minded” OR “Severely weak‐minded” OR “Multiply weak‐mindedness” OR “Profoundly weak‐mindedness” OR “Seriously weak‐mindedness” OR “Severely weak‐mindedness”) #2 (Alzheimer* OR Change OR Changed OR Changes OR Changing OR Decline OR Dement* OR Deteriorat* OR “Major cognitive dysfunction*” OR “Major cognitive impairment*” OR “Mild cognitive dysfunction*” OR “Mild cognitive impairment*” OR “Creutzfeldt Jakob” OR Degeneration OR Neurodegenerat* OR “Kluver‐Bucy” OR “Lewy Body” OR Cerebrovascular OR “Wilhelmsen Lynch” OR Pick* OR Binswanger OR Progressive OR Leukoencephalopathy OR Ageing OR Aging OR Neurodeteriorat* #3 Elder* OR Geriatric* OR “Old age” OR “Older participant*” OR “Older population*” OR “Older individual*” OR “Older adult*” OR “Older patient*” OR “Older people” OR “Older person*” OR “Older subject*” OR “Oldest participant*” OR “Oldest population*” OR “Oldest individual*” OR “Oldest adult*” OR “Oldest patient*” OR “Oldest people” OR “Oldest person*” OR “Oldest subject*” OR Senior* OR “Aged individual*” OR “Aged population*” OR “Aged participant*” OR “Aged adult*” OR “Aged patient*” OR “Aged people” OR “Aged person*” OR “Aged subject*” #4 TS = (#1) AND TS = (#2) AND TS = (#3)
PsycINFO	#1 Complex developmental abnormalit* OR Multiple developmental abnormalit* OR Profound developmental abnormalit* OR Serious developmental abnormalit* OR Severe developmental abnormalit* OR Complex intellectual abnormalit* OR Multiple intellectual abnormalit* OR Profound intellectual abnormalit* OR Serious intellectual abnormalit* OR Severe intellectual abnormalit* OR Complex learning abnormalit* OR Multiple learning abnormalit* OR Profound learning abnormalit* OR Serious learning abnormalit* OR Severe learning abnormalit* OR Complex mental abnormalit* OR Multiple mental abnormalit* OR Profound mental abnormalit* OR Serious mental abnormalit* OR Severe mental abnormalit* OR Complex neurodevelopmental abnormalit* OR Multiple neurodevelopmental abnormalit* OR Profound neurodevelopmental abnormalit* OR Serious neurodevelopmental abnormalit* OR Severe neurodevelopmental abnormalit* OR Amentia OR Complex cognitive challenge* OR Multiple cognitive challenge* OR Profound cognitive challenge* OR Serious cognitive challenge* OR Severe cognitive challenge* OR Complex developmental challenge* OR Multiple developmental challenge* OR Profound developmental challenge* OR Serious developmental challenge* OR Severe developmental challenge* OR Complex intellectual challenge* OR Multiple intellectual challenge* OR Profound intellectual challenge* OR Serious intellectual challenge* OR Severe intellectual challenge* OR Complex learning challenge* OR Multiple learning challenge* OR Profound learning challenge* OR Serious learning challenge* OR Severe learning challenge* OR Complex neurodevelopmental challenge* OR Multiple neurodevelopmental challenge* OR Profound neurodevelopmental challenge* OR Serious neurodevelopmental challenge* OR Severe neurodevelopmental challenge* OR Multiply cognitively challenged OR Profoundly cognitively challenged OR Seriously cognitively challenged OR Severely cognitively challenged OR Multiply developmentally challenged OR Profoundly developmentally challenged OR Seriously developmentally challenged OR Severely developmentally challenged OR Multiply intellectually challenged OR Profoundly intellectually challenged OR Seriously intellectually challenged OR Severely intellectually challenged OR Multiply learning challenged OR Profoundly learning challenged OR Seriously learning challenged OR Severely learning challenged OR Multiply neurodevelopmentally challenged OR Profoundly neurodevelopmentally challenged OR Seriously neurodevelopmentally challenged OR Severely neurodevelopmentally challenged OR Complex cognitive defect* OR Multiple cognitive defect* OR Profound cognitive defect* OR Serious cognitive defect* OR Severe cognitive defect* OR Complex developmental defect* OR Multiple developmental defect* OR Profound developmental defect* OR Serious developmental defect* OR Severe developmental defect* OR Complex intellectual defect* OR Multiple intellectual defect* OR Profound intellectual defect* OR Serious intellectual defect* OR Severe intellectual defect* OR Complex learning defect* OR Multiple learning defect* OR Profound learning defect* OR Serious learning defect* OR Severe learning defect* OR Complex mental defect* OR Multiple mental defect* OR Profound mental defect* OR Serious mental defect* OR Severe mental defect* OR Complex neurodevelopmental defect* OR Multiple neurodevelopmental defect* OR Profound neurodevelopmental defect* OR Serious neurodevelopmental defect* OR Severe neurodevelopmental defect* OR Multiply cognitively defective OR Profoundly cognitively defective OR Seriously cognitively defective OR Severely cognitively defective OR Multiply developmentally defective OR Profoundly developmentally defective OR Seriously developmentally defective OR Severely developmentally defective OR Multiply intellectually defective OR Profoundly intellectually defective OR Seriously intellectually defective OR Severely intellectually defective OR Multiply learning defective OR Profoundly learning defective OR Seriously learning defective OR Severely learning defective OR Multiply mentally defective OR Profoundly mentally defective OR Seriously mentally defective OR Severely mentally defective OR Multiply neurodevelopmentally defective OR Profoundly neurodevelopmentally defective OR Seriously neurodevelopmentally defective OR Severely neurodevelopmentally defective OR Complex cognitive deficienc* OR Multiple cognitive deficienc* OR Profound cognitive deficienc* OR Serious cognitive deficienc* OR Severe cognitive deficienc* OR Complex developmental deficienc* OR Multiple developmental deficienc* OR Profound developmental deficienc* OR Serious developmental deficienc* OR Severe developmental deficienc* OR Complex intellectual deficienc* OR Multiple intellectual deficienc* OR Profound intellectual deficienc* OR Serious intellectual deficienc* OR Severe intellectual deficienc* OR Complex learning deficienc* OR Multiple learning deficienc* OR Profound learning deficienc* OR Serious learning deficienc* OR Severe learning deficienc* OR Complex mental deficienc* OR Multiple mental deficienc* OR Profound mental deficienc* OR Serious mental deficienc* OR Severe mental deficienc* OR Complex neurodevelopmental deficienc* OR Multiple neurodevelopmental deficienc* OR Profound neurodevelopmental deficienc* OR Serious neurodevelopmental deficienc* OR Severe neurodevelopmental deficienc* OR Multiply cognitively deficient OR Profoundly cognitively deficient OR Seriously cognitively deficient OR Severely cognitively deficient OR Multiply developmentally deficient OR Profoundly developmentally deficient OR Seriously developmentally deficient OR Severely developmentally deficient OR Multiply intellectually deficient OR Profoundly intellectually deficient OR Seriously intellectually deficient OR Severely intellectually deficient OR Multiply learning deficient OR Profoundly learning deficient OR Seriously learning deficient OR Severely learning deficient OR Multiply mentally deficient OR Profoundly mentally deficient OR Seriously mentally deficient OR Severely mentally deficient OR Multiply neurodevelopmentally deficient OR Profoundly neurodevelopmentally deficient OR Seriously neurodevelopmentally deficient OR Severely neurodevelopmentally deficient OR Complex developmental deficit* OR Multiple developmental deficit* OR Profound developmental deficit* OR Serious developmental deficit* OR Severe developmental deficit* OR Complex intellectual deficit* OR Multiple intellectual deficit* OR Profound intellectual deficit* OR Serious intellectual deficit* OR Severe intellectual deficit* OR Complex learning deficit* OR Multiple learning deficit* OR Profound learning deficit* OR Serious learning deficit* OR Severe learning deficit* OR Complex mental deficit* OR Multiple mental deficit* OR Profound mental deficit* OR Serious mental deficit* OR Severe mental deficit* OR Complex neurodevelopmental deficit* OR Multiple neurodevelopmental deficit* OR Profound neurodevelopmental deficit* OR Serious neurodevelopmental deficit* OR Severe neurodevelopmental deficit* OR Complex cognitive delay OR Multiple cognitive delay OR Profound cognitive delay OR Serious cognitive delay OR Severe cognitive delay OR Complex developmental delay OR Multiple developmental delay OR Profound developmental delay OR Serious developmental delay OR Severe developmental delay OR Complex intellectual delay OR Multiple intellectual delay OR Profound intellectual delay OR Serious intellectual delay OR Severe intellectual delay OR Complex learning delay OR Multiple learning delay OR Profound learning delay OR Serious learning delay OR Severe learning delay OR Complex mental delay OR Multiple mental delay OR Profound mental delay OR Serious mental delay OR Severe mental delay OR Complex neurodevelopmental delay OR Multiple neurodevelopmental delay OR Profound neurodevelopmental delay OR Serious neurodevelopmental delay OR Severe neurodevelopmental delay OR Multiply cognitively delayed OR Profoundly cognitively delayed OR Seriously cognitively delayed OR Severely cognitively delayed OR Multiply developmentally delayed OR Profoundly developmentally delayed OR Seriously developmentally delayed OR Severely developmentally delayed OR Multiply intellectually delayed OR Profoundly intellectually delayed OR Seriously intellectually delayed OR Severely intellectually delayed OR Multiply learning delayed OR Profoundly learning delayed OR Seriously learning delayed OR Severely learning delayed OR Multiply mentally delayed OR Profoundly mentally delayed OR Seriously mentally delayed OR Severely mentally delayed OR Multiply neurodevelopmentally delayed OR Profoundly neurodevelopmentally delayed OR Seriously neurodevelopmentally delayed OR Severely neurodevelopmentally delayed OR Multiply differently‐abled OR Profoundly differently‐abled OR Seriously differently‐abled OR Severely differently‐abled OR Multiple developmental difficult* OR Complex developmental difficult* OR Profound developmental difficult* OR Serious developmental difficult* OR Severe developmental difficult* OR Multiple intellectual difficult* OR Complex intellectual difficult* OR Profound intellectual difficult* OR Serious intellectual difficult* OR Severe intellectual difficult* OR Complex learning difficult* OR Multiple learning difficult* OR Profound learning difficult* OR Serious learning difficult* OR Severe learning difficult* OR Multiple mental difficult* OR Complex mental difficult* OR Profound mental difficult* OR Serious mental difficult* OR Severe mental difficult* OR Complex neurodevelopmental difficult* OR Multiple neurodevelopmental difficult* OR Profound neurodevelopmental difficult* OR Serious neurodevelopmental difficult* OR Severe neurodevelopmental difficult* OR Complex cognitive disabilit* OR Multiple cognitive disabilit* OR Profound cognitive disabilit* OR Serious cognitive disabilit* OR Severe cognitive disabilit* OR Complex developmental disabilit* OR Multiple developmental disabilit* OR Multiple developmental disabilit* OR Serious developmental disabilit* OR Severe developmental disabilit* OR Trainable intellectual disabilit* OR Complex intellectual disabilit* OR Multiple intellectual disabilit* OR Profound intellectual disabilit* OR Serious intellectual disabilit* OR Severe intellectual disabilit* OR Severe profound intellectual motor disabilit* OR Complex learning disabilit* OR Multiple learning disabilit* OR Profound learning disabilit* OR Serious learning disabilit* OR Severe learning disabilit* OR Complex mental disabilit* OR Multiple mental disabilit* OR Profound mental disabilit* OR Serious mental disabilit* OR Severe mental disabilit* OR Trainable mental disabilit* OR Complex neurodevelopmental disabilit* OR Multiple neurodevelopmental disabilit* OR Profound neurodevelopmental disabilit* OR Serious neurodevelopmental disabilit* OR Severe neurodevelopmental disabilit* OR Multiple disabilit* OR Multiply cognitively disabled OR Profoundly cognitively disabled OR Seriously cognitively disabled OR Severely cognitively disabled OR Multiply developmentally disabled OR Profoundly developmentally disabled OR Seriously developmentally disabled OR Severely developmentally disabled OR Trainable developmentally disabled OR Multiply intellectually disabled OR Profoundly intellectually disabled OR Seriously intellectually disabled OR Severely intellectually disabled OR Trainable intellectually disabled OR Multiply learning disabled OR Profoundly learning disabled OR Seriously learning disabled OR Severely learning disabled OR Multiply mentally disabled OR Profoundly mentally disabled OR Seriously mentally disabled OR Severely mentally disabled OR Multiply neurodevelopmentally disabled OR Profoundly neurodevelopmentally disabled OR Seriously neurodevelopmentally disabled OR Severely neurodevelopmentally disabled OR Multiply disabled OR Trainable mentally disabled OR Complex developmental disorder* OR Multiple developmental disorder* OR Profound developmental disorder* OR Serious developmental disorder* OR Severe developmental disorder* OR Complex intellectual disorder* OR Multiple intellectual disorder* OR Profound intellectual disorder* OR Serious intellectual disorder* OR Severe intellectual disorder* OR Complex learning disorder* OR Multiple learning disorder* OR Profound learning disorder* OR Serious learning disorder* OR Severe learning disorder* OR Complex neurodevelopmental disorder* OR Multiple neurodevelopmental disorder* OR Profound neurodevelopmental disorder* OR Serious neurodevelopmental disorder* OR Severe neurodevelopmental disorder* OR Profoundly feeble‐minded OR Seriously feeble‐minded OR Severely feeble‐minded OR Profound feeble‐mindedness OR Serious feeble‐mindedness OR Severe feeble‐mindedness OR Complex cognitive handicap* OR Multiple cognitive handicap* OR Profound cognitive handicap* OR Serious cognitive handicap* OR Severe cognitive handicap* OR Complex developmental handicap* OR Multiple developmental handicap* OR Profound developmental handicap* OR Serious developmental handicap* OR Severe developmental handicap* OR Complex intellectual handicap* OR Multiple intellectual handicap* OR Profound intellectual handicap* OR Serious intellectual handicap* OR Severe intellectual handicap* OR Complex learning handicap* OR Multiple learning handicap* OR Profound learning handicap* OR Serious learning handicap* OR Severe learning handicap* OR Complex mental handicap* OR Multiple mental handicap* OR Profound mental handicap* OR Serious mental handicap* OR Severe mental handicap* OR Trainable mental handicap* OR Complex neurodevelopmental handicap* OR Multiple neurodevelopmental handicap* OR Profound neurodevelopmental handicap* OR Serious neurodevelopmental handicap* OR Severe neurodevelopmental handicap* OR Multiply cognitively handicapped OR Profoundly cognitively handicapped OR Seriously cognitively handicapped OR Severely cognitively handicapped OR Multiply developmentally handicapped OR Profoundly developmentally handicapped OR Seriously developmentally handicapped OR Severely developmentally handicapped OR Multiply intellectually handicapped OR Multiply intellectually handicapped OR Profoundly intellectually handicapped OR Profoundly intellectually handicapped OR Seriously intellectually handicapped OR Seriously intellectually handicapped OR Severely intellectually handicapped OR Severely intellectually handicapped OR Multiply learning handicapped OR Profoundly learning handicapped OR Seriously learning handicapped OR Severely learning handicapped OR Multiply mentally handicapped OR Profoundly mentally handicapped OR Seriously mentally handicapped OR Severely mentally handicapped OR Multiply neurodevelopmentally handicapped OR Profoundly neurodevelopmentally handicapped OR Seriously neurodevelopmentally handicapped OR Severely neurodevelopmentally handicapped OR Multiply handicapped OR Trainable mentally handicapped OR Idiocy OR Idiot OR Idiotcy OR Idiotic OR Idiotism OR Idiots OR Imbecil* OR Multiply developmentally impaired OR Profoundly developmentally impaired OR Seriously developmentally impaired OR Severely developmentally impaired OR Multiply intellectually impaired OR Profoundly intellectually impaired OR Seriously intellectually impaired OR Severely intellectually impaired OR Multiply learning impaired OR Profoundly learning impaired OR Seriously learning impaired OR Severely learning impaired OR Multiply mentally impaired OR Profoundly mentally impaired OR Seriously mentally impaired OR Severely mentally impaired OR Multiply neurodevelopmentally impaired OR Profoundly neurodevelopmentally impaired OR Seriously neurodevelopmentally impaired OR Severely neurodevelopmentally impaired OR Trainable mentally impaired OR Complex developmental impairment* OR Multiple developmental impairment* OR Profound developmental impairment* OR Serious developmental impairment* OR Severe developmental impairment* OR Complex intellectual impairment* OR Multiple intellectual impairment* OR Profound intellectual impairment* OR Serious intellectual impairment* OR Severe intellectual impairment* OR Complex learning impairment* OR Multiple learning impairment* OR Profound learning impairment* OR Serious learning impairment* OR Severe learning impairment* OR Complex mental impairment* OR Multiple mental impairment* OR Profound mental impairment* OR Serious mental impairment* OR Severe mental impairment* OR Complex neurodevelopmental impairment* OR Multiple neurodevelopmental impairment* OR Profound neurodevelopmental impairment* OR Serious neurodevelopmental impairment* OR Severe neurodevelopmental impairment* OR Trainable mental impairment* OR Complex cognitive incapacit* OR Multiple cognitive incapacit* OR Profound cognitive incapacit* OR Serious cognitive incapacit* OR Severe cognitive incapacit* OR Complex developmental incapacit* OR Multiple developmental incapacit* OR Profound developmental incapacit* OR Serious developmental incapacit* OR Severe developmental incapacit* OR Complex intellectual incapacit* OR Multiple intellectual incapacit* OR Profound intellectual incapacit* OR Serious intellectual incapacit* OR Severe intellectual incapacit* OR Complex learning incapacit* OR Multiple learning incapacit* OR Profound learning incapacit* OR Serious learning incapacit* OR Severe learning incapacit* OR Complex mental incapacit* OR Multiple mental incapacit* OR Profound mental incapacit* OR Serious mental incapacit* OR Severe mental incapacit* OR Complex neurodevelopmental incapacit* OR Multiple neurodevelopmental incapacit* OR Profound neurodevelopmental incapacit* OR Serious neurodevelopmental incapacit* OR Severe neurodevelopmental incapacit* OR Multiply cognitively incapacitated OR Profoundly cognitively incapacitated OR Seriously cognitively incapacitated OR Severely cognitively incapacitated OR Multiply developmentally incapacitated OR Profoundly developmentally incapacitated OR Seriously developmentally incapacitated OR Severely developmentally incapacitated OR Multiply intellectually incapacitated OR Profoundly intellectually incapacitated OR Seriously intellectually incapacitated OR Severely intellectually incapacitated OR Multiply learning incapacitated OR Profoundly learning incapacitated OR Seriously learning incapacitated OR Severely learning incapacitated OR Multiply mentally incapacitated OR Profoundly mentally incapacitated OR Seriously mentally incapacitated OR Severely mentally incapacitated OR Multiply neurodevelopmentally incapacitated OR Profoundly neurodevelopmentally incapacitated OR Seriously neurodevelopmentally incapacitated OR Severely neurodevelopmentally incapacitated OR Polyhandicap* OR Trainable mentally retardate* OR Complex cognitive retardation OR Multiple cognitive retardation OR Profound cognitive retardation OR Serious cognitive retardation OR Severe cognitive retardation OR Complex developmental retardation OR Multiple developmental retardation OR Profound developmental retardation OR Serious developmental retardation OR Severe developmental retardation OR Complex intellectual retardation OR Multiple intellectual retardation OR Profound intellectual retardation OR Serious intellectual retardation OR Severe intellectual retardation OR Complex learning retardation OR Multiple learning retardation OR Profound learning retardation OR Serious learning retardation OR Severe learning retardation OR Complex mental retardation OR Multiple mental retardation OR Profound mental retardation OR Serious mental retardation OR Severe mental retardation OR Trainable mental retardation OR Complex neurodevelopmental retardation OR Multiple neurodevelopmental retardation OR Profound neurodevelopmental retardation OR Serious neurodevelopmental retardation OR Severe neurodevelopmental retardation OR Multiply cognitively retarded OR Profoundly cognitively retarded OR Seriously cognitively retarded OR Severely cognitively retarded OR Multiply developmentally retarded OR Profoundly developmentally retarded OR Seriously developmentally retarded OR Severely developmentally retarded OR Multiply intellectually retarded OR Profoundly intellectually retarded OR Seriously intellectually retarded OR Severely intellectually retarded OR Multiply learning retarded OR Profoundly learning retarded OR Seriously learning retarded OR Severely learning retarded OR Multiply mentally retarded OR Profoundly mentally retarded OR Seriously mentally retarded OR Severely mentally retarded OR Multiply neurodevelopmentally retarded OR Profoundly neurodevelopmentally retarded OR Seriously neurodevelopmentally retarded OR Severely neurodevelopmentally retarded OR Trainable mentally retarded OR Trainable retarded OR Multiply cognitively subnormal OR Profoundly cognitively subnormal OR Seriously cognitively subnormal OR Severely cognitively subnormal OR Multiply developmentally subnormal OR Profoundly developmentally subnormal OR Seriously developmentally subnormal OR Severely developmentally subnormal OR Multiply intellectually subnormal OR Profoundly intellectually subnormal OR Seriously intellectually subnormal OR Severely intellectually subnormal OR Multiply learning subnormal OR Profoundly learning subnormal OR Seriously learning subnormal OR Severely learning subnormal OR Multiply mentally subnormal OR Profoundly mentally subnormal OR Seriously mentally subnormal OR Severely mentally subnormal OR Multiply neurodevelopmentally subnormal OR Profoundly neurodevelopmentally subnormal OR Seriously neurodevelopmentally subnormal OR Severely neurodevelopmentally subnormal OR Multiply weak‐minded OR Profoundly weak‐minded OR Seriously weak‐minded OR Severely weak‐minded OR Multiply weak‐mindedness OR Profoundly weak‐mindedness OR Seriously weak‐mindedness OR Severely weak‐mindedness #2 Alzheimer* OR Change OR Changed OR Changes OR Changing OR Decline OR Dement* OR Deteriorat* OR Major cognitive dysfunction* OR Major cognitive impairment* OR Mild cognitive dysfunction* OR Mild cognitive impairment* OR Creutzfeldt Jakob OR Degeneration OR Neurodegenerat* OR K Kluver‐Bucy OR Lewy Body OR Cerebrovascular OR Wilhelmsen Lynch OR Pick* OR Binswanger OR Progressive OR Leukoencephalopathy OR Aging OR Aging OR Neurodeteriorat* #3 Elder* OR Geriatric* OR Old age OR Older participant* OR Older population* OR Older individual* OR Older adult* OR Older patient* OR Older people OR Older person* OR Older subject* OR Oldest participant* OR Oldest population* OR Oldest individual* OR Oldest adult* OR Oldest patient* OR Oldest people OR Oldest person* OR Oldest subject* OR Senior* OR Aged individual* OR Aged population* OR Aged participant* OR Aged adult* OR Aged patient* OR Aged people OR Aged person* OR Aged subject* #4 [#1 TI AND #2 TI AND #3 TI] OR [#1 AB AND #2 AB AND #3 AB]
